# Magmatic immiscibility provides phosphate for prebiotic chemistry

**DOI:** 10.1126/sciadv.adz2567

**Published:** 2025-11-19

**Authors:** Daniel Weller, Thomas Matreux, Iris B. A. Smokers, Almuth Schmid, Christof B. Mast, Donald B. Dingwell, Dieter Braun, Bettina Scheu

**Affiliations:** ^1^ Earth and Environmental Sciences, Ludwig Maximilians University, Munich, Germany.; ^2^ Laboratoire de Biophysique et Evolution, UMR CNRS-ESPCI 8231 Chimie Biologie Innovation, PSL University, Paris, France.; ^3^ Systems Biophysics, Ludwig Maximilians University, Munich, Germany.; ^4^ Institute for Molecules and Materials, Radboud University, Nijmegen, Netherlands.; ^5^ GEOLAB, Hangzhou International Innovation Institute, Beihang University, Hangzhou, China.

## Abstract

Phosphorus is essential for extant and nascent life. However, phosphate’s low terrestrial abundance and the poor aqueous solubility of phosphorus-bearing minerals pose major hurdles for prebiotic chemistry. Here, we show that silicate-phosphate immiscibility in volcanic melts could have provided a widely available mechanism for the local enrichment of phosphate. Starting with a phosphorus-enriched Archean melt, we observe that rapid cooling results in the formation of immiscible glassy droplets with up to 21 weight % phosphorus pentoxide, which can be delivered to the surface by volcanic processes. In aqueous leaching, we detect millimolar phosphate concentrations, which enable the synthesis of phosphorylating agents such as polyphosphates and imidazole phosphate with up to 34% yield. These results demonstrate how volcanic processes can enrich phosphate to fuel prebiotic chemistry.

## Teaser

Volcanic immiscibility provides a key mechanism to enrich phosphate, fueling prebiotic chemistry.

## INTRODUCTION

Phosphorus is crucial for modern biology in RNA, DNA, the universal energy carrier adenosine 5′-triphosphate (ATP), and in phospholipids. This role in numerous biochemical processes ultimately points to its central importance during the emergence of life. However, this poses a major problem for nascent life, as the utilization of phosphorus by prebiotic chemistry is hindered by its relative scarcity on early and modern Earth’s surface compared to the other elements of life and the low aqueous solubility of common phosphate minerals such as apatite (*1*–*3*). Experimental studies using apatite have shown that chelating agents such as oxalate or citrate can increase aqueous phosphate solubility (*4*) and that thermal nonequilibrium environments (*2*) and carbonate-rich lakes (*5*) can enrich phosphate to concentrations relevant for prebiotic chemistry. Other studies have focused on rare minerals such as struvite (*6*) or synthetic phosphate salts to circumvent this problem (*7*, *8*). Further possible sources of phosphorus include reduced species resulting from lightning strikes (*9*), thermal reduction in Fe^2+^-rich sediments (*10*), or extraterrestrial influx in the form of schreibersite (*11*). To date, however, a fundamental, universally accepted mechanism how early Earth could have provided phosphate for prebiotic chemistry remains unknown.

**Fig. 1.**
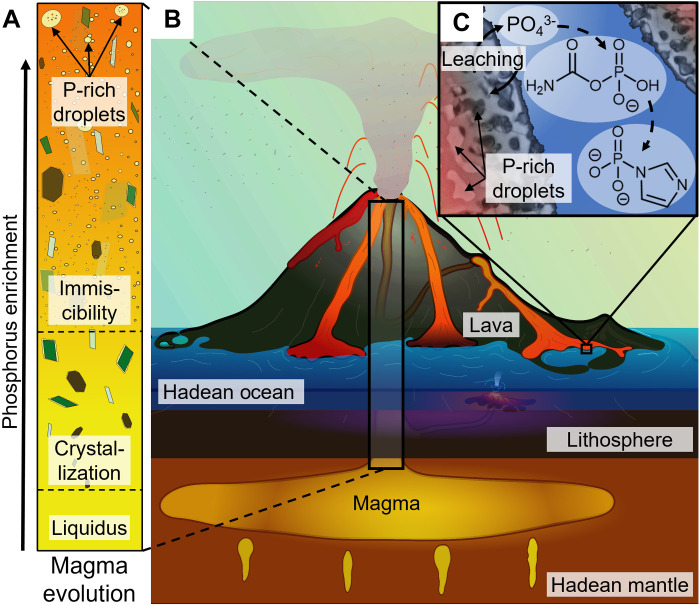
Enrichment and delivery of magmatic phosphate. (**A**) Magma ascent and unmixing during cooling. Upon ascent, the melt evolves from an initial crystal-poor, homogeneous silicate liquid, through magmatic differentiation and unmixing, into a multiphase system containing P-enriched immiscible droplets. (**B**) Schematic depiction of volcanic hadean environment. Upon eruption, the magma quenches in contact with air or water. (**C**) The glassy P-enriched droplets are leached in water to provide phosphate, driving the formation of various phosphorylating agents such as imidazole phosphate, crucial for prebiotic chemistry.

Here, we show that a previously unconsidered yet ubiquitous magmatic and volcanic phase transition could have provided a widely available phosphate source for the emergence of life. Despite its low P_2_O_5_ content of ~0.2 wt %, the mantle remains the main phosphorus reservoir of bulk silicate Earth (*12*), containing 95% of its phosphorus (*13*). During the melting of the mantle and subsequent magmatic evolution, for example at hot spot volcanism, the dilute phosphorus behaves as a geochemically incompatible element in early magmatic differentiation and mostly remains in the basaltic melt (*14*). This passive phosphorus enrichment can result in P_2_O_5_ concentrations up to 3 to 4 wt % without crystallization of apatite (*15*–*17*). As mafic melts migrate to the surface (Fig. 1A), they commonly reach a depth where pressure and temperature and melt and fluid compositions induce liquid immiscibility (*18*, *19*), whereby two coexisting liquids of distinct composition become thermodynamically stable. The extent of this so-called “two-liquid field” is substantially influenced by P_2_O_5_ and TiO_2_ concentrations (*20*, *21*). The resulting emulsion of P-enriched droplets in a Si-enriched matrix (*20*) is morphologically similar to other systems exhibiting liquid-liquid phase separation, such as metal alloys (*22*) or biomolecular condensates (*23*).

Evidence for liquid immiscibility has been observed in various igneous rocks (*24*–*29*), for example, as boundary layers around crystals (*27*) or within interstitial melt pockets (mesostasis) during late-stage crystallization (*25*). In the phosphorus-rich droplet phase, up to ~30 wt % P_2_O_5_ has been detected (*26*). The process has also been reproduced in synthetic samples under laboratory conditions (*15*, *20*, *30*–*33*), yielding concentrations of near 40 wt % P_2_O_5_ (*15*). Continuous enrichment through magmatic differentiation and droplet formation has been shown to eventually lead to the formation of Fe-P “slags” (fig. S1) (*34*) or apatite-rich deposits (*33*–*39*) when the droplets fractionate gravitationally. However, the droplets can also remain suspended in the mafic magma together with crystals and gas bubbles (Fig. 1B) during transport to the surface by volcanic activity (*24*, *25*, *40*). Here, the lava forms basalt, the presumably dominant type of volcanic rock on early Earth (*41*) and can be cooled rapidly enough in contact with water to form a glassy silicate rock (vitrophyric), e.g., at the chilled margins of pillow lavas and as hyaloclastites. This process is accompanied by fracturing due to thermal stresses, generating pathways for rock-fluid interaction. Via aqueous leaching, the glassy phases could then supply phosphate for prebiotic reactions (Fig. 1C).

In this study, we experimentally investigated unmixing in a synthetic tholeiitic glass, mimicking a phosphorus-enriched Archean composition. The glass was produced by rapidly cooling the mixture from 1600°C to room temperature (RT) and then chemically and texturally analyzed by scanning electron microscopy (SEM) and electron probe microanalysis. After confirming the glassy state of unmixed Fe-P rich droplets and the surrounding Si-rich matrix, we investigated the leaching behavior in deionized water over a range of pH, grain sizes, and chemical additives. To evaluate the usability of leached phosphate for prebiotic chemistry, we explored its potential to generate polyphosphates, phosphorylate nucleosides, and form imidazole phosphate.

## RESULTS

### Properties of phosphorus-rich immiscible basaltic glasses

Ample evidence from natural (*24*–*29*) and synthetic (*15*, *20*, *30*–*33*) samples demonstrates that late-stage basaltic unmixing can lead to strong local enrichment of phosphorus in an immiscible glassy phase. To mimic this process and evaluate its relevance for prebiotic chemistry in the absence of preserved terrestrial Hadean glasses, we used an average composition of Archean rocks with a P_2_O_5_ concentration of more than 0.5 wt % as basis for our synthetic sample (table S1) (*42*–*60*). For the presence of natural (endmember-composition) immiscible glassy droplets and to ensure the synthesis of a crystal-free glass, diammonium hydrogen phosphate was added to the mixture to achieve a bulk P_2_O_5_ content of 10 wt % (*15*) (see tables S1 and S2 for composition and Materials and Methods for details).

**Fig. 2.**
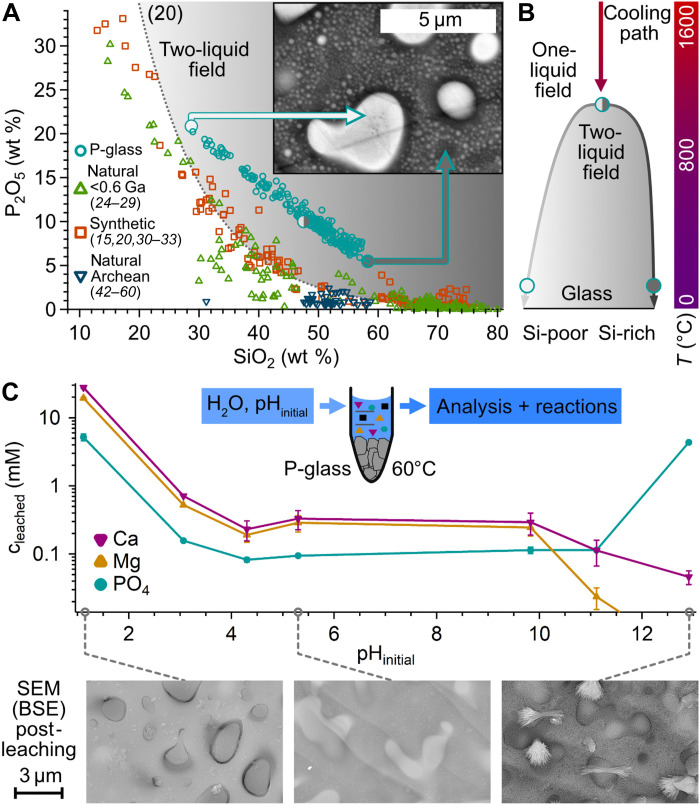
Formation and characterization of phosphate-bearing volcanic glass. (**A**) P_2_O_5_ concentrations (turquoise circles) are shown in the context of previous studies on natural (green; <0.6 Ga) (*24*–*29*), synthetic (orange) (*15*, *20*, *30*–*33*), and natural Archean (blue) (*42*–*60*) samples (see fig. S1 for individual references). Compositional extreme values of the P-glass are highlighted in the SEM-BSE image. (**B**) Schematic representation of the thermal evolution of a P-enriched system (*20*), indicating the cooling path of the P-glass. When reaching the two-liquid field, Fe-, P-, Ca-, Mg-enriched droplets (light color) nucleate and grow in an increasingly Si-, Al-rich matrix (dark color). (**C**) Phosphate leaching in water and surface alteration. The unmixed glass was incubated at pH_initial_ values for 72 hours at 60°C. Ion chromatography (IC) revealed leached concentrations (c_leached_) with up to 5 mM phosphate for acidic and basic conditions, driven by the preferential dissolution of P-enriched droplets (see SEM images and fig. S7). For other chemical solutions, pH buffering, agitation, and grain sizes, see figs. S8 to S12. All error bars show the SD.

The powder mixture was melted and held at 1600°C for 72 hours and subsequently quenched in a crucible to obtain a glassy state. The glassy product (P-glass) exhibited clear signs of liquid immiscibility [Fig. 2A; SEM–backscattered electron (BSE) image]. We characterize the two immiscible melts using the depolymerization index of nonbridging oxygens to tetrahedral cations after Mysen *et al.* (*61*) to visualize this petrological immiscibility (fig. S2) (*20*). When cooling from 1600°C, the homogeneous melt intersects the binodal curve of the two-liquid stability field whereupon it unmixes into Fe-, P-, Ca-, Mg-enriched droplets and a Si-, Al-enriched matrix. With rising viscosity under quenching conditions, the adjacent melts transition into glasses and remain as such upon cooling (Fig. 2B).

Liquid-liquid unmixing yields a wide range of size (<0.1 to 10 μm), shape, and chemical composition of droplets (Fig. 2A, figs. S3 and S4, and table S3), measured by electron probe microanalysis (EPMA) combined with SEM. The amorphous character of droplets and matrix was verified with powder x-ray diffraction (XRD) (fig. S5). While the droplets have P_2_O_5_ concentrations of up to 20.8 wt % (Fig. 2A), we observe 5.3 to 10.0 wt % P_2_O_5_ in the Si-enriched matrix. Observed SiO_2_ and P_2_O_5_ concentrations (Fig. 2A) are similar to those found in natural immiscible samples (*24*–*29*). The concentration ratio of phosphorus $D_{P}=\frac{{\left[P_{2}O_{5}\right]}_{\text{max}}}{{\left[P_{2}O_{5}\right]}_{\text{min}}}=3.9$ (fig. S6) is higher than the ratio of coenriched cations *D*_Fe_ = 3.0, *D*_Mg_ = 2.9, and *D*_Ca_ = 2.5 (fig. S3).

### Leaching of phosphate

Key prebiotic compounds such as RNA, ATP, and lipids contain phosphates, representing a massive requirement for dissolved phosphate for nascent life. We tested the phosphate release from the P-glass by analyzing the leached ion compositions for a wide range of initial pH values in deionized water (Fig. 2C and Materials and Methods). Natural acidic and basic pH settings (*62*) yielded up to 5.2 and 4.4 mM phosphate, respectively, presumably driven by the preferential dissolution of P-enriched droplets. Ca and Mg concentrations follow a similar pH dependency toward acidic pH values but decrease at alkaline pH. For intermediary acidic or basic environments, rock-water interaction led to buffering toward neutral pH values (fig. S10) and caused partial reprecipitation (as visible in Fig. 2C and fig. S7; SEM images), limiting dissolved ion concentrations. Smaller grain sizes showed an increased phosphate release, explained by a larger surface area (fig. S8). For very small grains (<32 μm), wetting becomes incomplete, limiting phosphate concentrations (*63*). Agitation during incubation had no impact on leached concentrations (fig. S11). Addition of citrate, which removes calcium as binding partner (*64*), showed a strong boost in leached phosphate concentrations, up to 3 mM at pH 4 (fig. S12). Neither carbonate, which also removes calcium as binding partner (*5*), nor ammonia and urea, which facilitate phosphorylation (*4*, *7*, *65*), had a substantial effect on phosphate leaching. Experiments in artificial sea water (*63*, *66*) show slightly lower leached phosphate concentrations, presumably due to reprecipitation (fig. S12).

To exemplify the direct usage of leached phosphate for prebiotic chemistry, we benchmarked the phosphorylation of the RNA nucleoside adenosine. For this, we added adenosine to the leachate and heated the sample to 60°C over 72 hours in open vials to induce a wet-to-dry transition, resulting in the formation of adenosine 5′-monophosphate with a yield of up to 14 nmol/g of basaltic glass, corresponding to a yield of 0.4% relative to supplemented adenosine (fig. S13 and Materials and Methods). Yields were more than twofold higher when the experiment was done in the presence of P-glass compared to when it was removed before adenosine addition. As discussed in literature (*7*, *8*), the low yield illustrates the limited reactivity of orthophosphate for phosphorylation reactions in water.

### Synthesis of phosphorylating agents

**Fig. 3.**
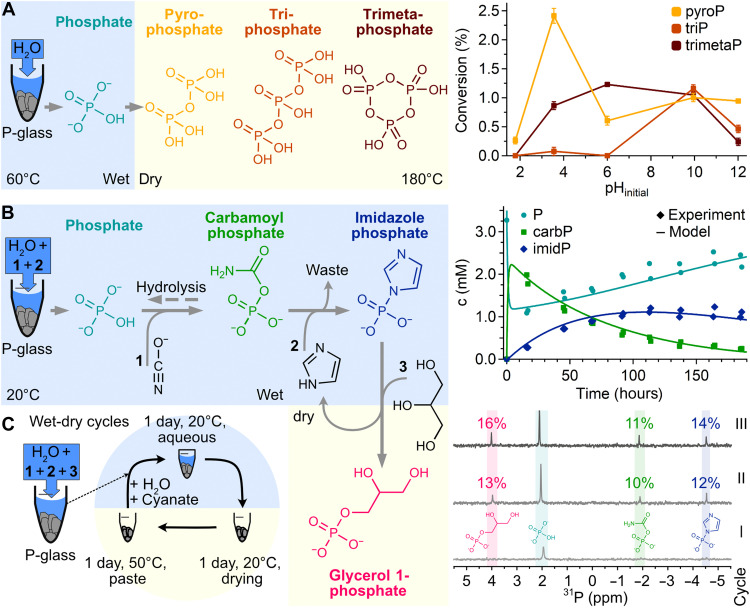
Fueling the synthesis of activated phosphate species and prebiotic phosphorylation. (**A**) Formation of polyphosphates from leached phosphate (cyan) upon heating. Heating over 72 hours at 180°C yields pyrophosphate (yellow) from acidic leachates and a mixture of condensed phosphate species at neutral and basic pH values. pyroP, pyrophosphate; triP, triphosphate; trimetaP, trimetaphosphate. (**B**) Activation of leached phosphate to form the phosphorylating agent imidazole phosphate (blue). After 48 hours of leaching in imidazole (2) buffer, addition of cyanate (1) induces the formation of carbamoyl phosphate (green) and imidazole phosphate from orthophosphate in aqueous solution. A maximum yield of 34% imidazole phosphate is obtained after 115 hours. Results are similar when P-glass is removed before cyanate addition (fig. S15). Lines show fitted reaction kinetics (Materials and Methods). p, phosphate; carbP, carbamoyl phosphate; imidP, imidazole phosphate. (**C**) Phosphate transfer to glycerol. Wet-dry cycles facilitate subsequent phosphorylation of glycerol (3) to form the phospholipid-precursor glycerol phosphate. After 3 cycles, 16.5% of leached phosphate is transferred to glycerol 1-phosphate (pink), as shown by ^31^P-NMR (*74*). All error bars show the SD.

The poor reactivity of orthophosphate has driven research toward more reactive phosphate species such as prebiotically relevant polyphosphates. These were shown to be capable of various reactions including the ligation of amino acids (*67*–*70*) and have also been found in volcanic outgassing environments (*71*). To explore the direct conversion of the orthophosphate obtained from P-glass to more active polyphosphates, we dried the leachates at 180°C over 72 hours (*2*, *72*) and redissolved the product in H_2_O for analysis by ion chromatography (IC; Materials and Methods). While acidic initial pH values reduced overall efficiency of phosphate polymerization, up to 18 nmol pyrophosphate per gram basaltic glass (fig. S14) were formed, corresponding to a conversion of 2.5% with respect to the total amount of leached phosphate (Fig. 3A). Linear triphosphates were detected for initial pH values of 10 or above, while up to 10 nmol/g of trimetaphosphate, corresponding to 1.2% of the leached phosphate, were observed for initial pH values between 3 and 12. Observed redissolved yields were lower when samples were heated in the presence of P-glass, presumably due to adsorption and surface interactions (fig. S14).

Another phosphorylating agent shown to overcome phosphate’s poor reactivity for a wide range of substrates is imidazole phosphate. It can be formed from phosphate in aqueous solution by reaction with cyanate (*73*) and imidazole (*74*–*77*) under mild and pH-neutral conditions (Fig. 3B; see text S1 for an extended discussion of the reagents). We investigated the efficiency of capturing phosphate leached from P-glass in imidazole phosphate by immersing P-glass particles for 48 hours at 20°C in an imidazole solution (Materials and Methods). Hence, we could probe the effect of P-glass on the reaction by either using only the leachate or the rock-leachate suspension. The addition of potassium cyanate started the reaction, which was analyzed by ^31^P–nuclear magnetic resonance (NMR) spectroscopy and quantified with an internal standard (*74*). The rapidly formed carbamoyl phosphate reacted further to form the more stable imidazole phosphate, captured by fitted reaction kinetics (Eqs. 1 to 5 and Materials and Methods). Final concentrations of imidazole phosphate reached up to 1.2 mM, corresponding to a conversion of 34% of the leached phosphate (Fig. 3B) in the presence of P-glass and 28% in its absence during the reaction (fig. S15).

Once formed, imidazole phosphate can phosphorylate a wide variety of substrates (*74*). We probed the phosphorylation of glycerol to form the (phospho-)lipid–precursor glycerol phosphate (Fig. 3C) from volcanic P-enriched glass by adding imidazole, potassium cyanate, and glycerol (*74*) to the P-glass. After incubation for 1 day at 20°C, leading to the formation of imidazole phosphate (as before), the leachate-rock suspension was exposed to wet-dry cycles, as it can occur in prebiotic ponds that dry in the sun and are refilled by rain or tides. For this, the suspension of P-glass and leachate was dried to a paste for 1 day at 20°C, heated at 50°C for 1 day, and redissolved in potassium cyanate to repeat the cycle (see Fig. 3C and Materials and Methods for details). After 3 wet-dry cycles, 16.5% of leached phosphate was converted to glycerol 1-phosphate. To see whether the line broadening by paramagnetic Fe^2+^ in ^31^P-NMR lead to an underestimation of formed product, we treated the dissolved sample with cation exchange resin. This confirmed the conversion yield of glycerol 1-phosphate and showed glycerol 2-phosphate as a minor product with 2.8% conversion yield (figs. S16 to S19; after a fourth cycle) as expected from literature (*74*).

## DISCUSSION

Magmatic unmixing by liquid immiscibility could hold a substantial yet largely unexplored key to locally enrich phosphorus and promote its chemical accessibility for nascent life. Today, immiscible globules are found in a wide range of settings and melt compositions in the shape of mesostasis, as inclusions, around crystals, or dispersed in evolved basaltic rocks (*24*, *25*) transported from various depths in the mantle. In natural Phanerozoic samples, these range from 7 to 8 wt % in the Umtanum formation (*29*) and on Kerguelen Island (*25*) to 30.2 wt % P_2_O_5_ in Fe-, Ti-, Ca-, P-rich globules in Siberian Trap gabbros (*26*). Other volcanic examples include Hawaii, Iceland, and the island of Mull, as well as large effusive events that formed the Snake River Plain and layered intrusions of Skaergaard, Sept Iles, and Bushveld. Some models even propose widespread unmixing at mid-ocean ridges and in the lower oceanic crust (*78*).

Evidence of magmatic unmixing has been found in Archean basalts (*79*, *80*) and lunar rocks preserved from volcanic activity during its formation (*81*), implying that similar processes could have occurred on early Earth, rendering P-rich immiscible melt droplets accessible for prebiotic chemistry. A high degree of melting, in combination with slow cooling and advanced stages of magmatic evolution, as, for example, found in hot spot–related volcanic systems such as Hawaii, could produce a melt enriched in phosphorus. The higher magmatic and eruptive temperatures in the Hadean (*82*) would have negatively affected phosphorus incorporation in olivine (*83*, *84*), while apatite solubility would have been increased (fig. S20) (*15*). This could have amplified the importance of phosphorus accumulation via immiscibility, favoring the formation of phosphorus-rich glasses on the surface. Unmixed globules can form a glass depending on cooling rates, for instance, after eruption in direct contact with air, water, or ice.

The speciation and redox state of the magmatic phosphorus transported from a (deep) mantle source and the influence of liquid immiscibility in this process require further investigation. In addition, a study on strongly different mantle and surface temperatures as in the Hadean and the coreduction of carbon, iron, and phosphorus to form Fe_3_P melt pools (*34*) could be relevant for the origin of life.

We used phosphate-doped Archean basalt in this study, yet the immiscible Fe-P droplets are very similar to those found in nature. Actual phosphorus concentrations in Hadean immiscible rocks remain speculative, yet remnant bulk tholeiitic basalts from the Archean were found to carry up to 2 wt % P_2_O_5_ (fig. S20). As these rocks are billions of years old and have been subject to weathering, geochemical investigations using P/Zr ratios could be useful for estimating the original phosphorus concentrations (*85*). While natural immiscible glasses can show lower P_2_O_5_ contents, the increased solubility of the droplets presumably remains, and absolute concentrations could have been increased by drying (*5*, *8*) and thermophoretic accumulation (*2*, *62*, *63*, *68*).

Immiscible droplets, such as those in our synthetic samples, leach elevated concentrations of phosphorus. Although the synthesized P-glass has four times lower bulk P_2_O_5_ content than the pure phosphate mineral apatite, the amount of leached phosphate at neutral pH (0.1 mM) exceeds comparable experiments with apatite by more than one order of magnitude (~0.005 mM; fig. S21). We benchmarked the prebiotic usage of this magmatic phosphate supply by investigating the aqueous leaching of the unmixed sample, as well as the formation of condensed phosphate (*65*, *69*, *86*, *87*) and chemically activated species such as carbamoyl and imidazole phosphates (*74*, *88*, *89*). We could show that polyphosphates can reach similar yields as in previous experiments at 200°C and controlled H_2_ atmosphere without the presence of a catalysts (*72*), while an excess of Fe^2+^ as catalyst (*72*) and apatite constituents (*2*) was shown to increase yields. Furthermore, the transfer of phosphate from the P-enriched glassy droplets to prebiotically relevant molecules such as glycerol gave similar yields as in previous studies (*74*).

Phosphorus enrichment by unmixing in basaltic melts has to date not been considered in the context of the emergence of life. Here, we show how magmatic sources could offer a notable contribution by exploiting Earth’s primary phosphorus reservoir without the need for extraterrestrial phosphate sources. The observed local accumulation of phosphorus by liquid immiscibility illustrates how Hadean volcanic environments could have satisfied the phosphate hunger of prebiotic chemistry.

## MATERIALS AND METHODS

### Preparation of phosphorus-rich glass (P-glass)

Oxides (SiO_2_, Al_2_O_3_, TiO_2_, Fe_2_O_3_/Fe_3_O_4_, and MgO), carbonates (CaCO_3_, NaCO_3_, and K_2_CO_3_), and phosphate ((NH_4_)_2_HPO_4_) of high purity were purchased from VWR and Th. Geyer and weighed with a high-precision mass balance for 10 wt % P_2_O_5_ content (table S2). The mixture was manually homogenized in an agate mortar. A thin-walled platinum crucible (75 cm^3^) was used to melt the mixture at 1550° to 1600°C in a MoSi_2_ box furnace in atmosphere for 48 hours to ensure a homogeneous and crystal free melt. The liquid was cooled to RT without any further quenching, as viscosity was too high for pouring. The resulting glass was carefully crushed and sieved for selected grain sizes (e.g., 355 to 500 μm).

### EPMA

EPMA were conducted at Ludwig Maximilians University Munich (LMU) with a CAMECA SX100 EPMA system equipped with a LaB6 cathode and wavelength-dispersive x-ray spectrometers. Analytical conditions were set to 15-keV acceleration voltage, with 5-nA emission current, and a 10-μm defocused beam. Spectrometers were calibrated with albite (Na, Si), periclase (Mg), orthoclase (Al, K), apatite (P, Ca), ilmenite (Ti), and hematite syn. (Fe) as standards. Glass shards were measured with 60 points each. Averages and standard deviations are given in table S3. EPMA measurements of droplets proofed challenging, as the droplet size ranged from ≪0.1 to 15 μm with very-high–number densities (*25*). Even with a focused beam, it was difficult to distinguish the Si-rich phase surrounding the Fe-rich droplets in the glass.

### Scanning electron microscopy

A Hitachi SU-5000 at LMU was used in BSE-EDS mode for imaging and chemical analysis of glass particles before and after leaching experiments. Conditions were set to 20-kV acceleration voltage, 177-μA emission current, and 11-mm working distance. Samples were either measured as loose particles or polished epoxy mounts, both coated with ~13- to 14-nm carbon.

### Volume and density distribution analysis by fast object acquisition and measurement system

BSE images of polished P-glass samples captured with the SEM were binarized with ImageJ and analyzed with fast object acquisition and measurement system, a MATLAB-based program usually applied in geoscience to quantitatively and qualitatively measure spherical objects such as vesicles (*90*), which can be applied for particles or droplets. Volumetric proportions and size distributions are calculated by stereographical two-dimensional (2D) to 3D conversion (*91*), and corrections for intersecting spheres or elliptical shapes are automatically applied. Methodology can be compared with (*92*). Single images were measured, and resulting volume fractions of the Fe-P–enriched droplets were calculated from 2D integration.

### X-ray diffraction

The glass was manually ground in an agate mortar to particle size < 63 μm and filled into a glass capillary (0.5 mm Ø), which was, in return, mounted to a goniometer. The sample was measured in an XRD 3003 TT diffractometer at RT and stepscan mode (width: 0.013°) for 120 min using Mo Kα_1_ radiation (40-mA emission current and 40-kV voltage). 2θ angles of 4.5° to 60° were considered for interpretation. Curvature and possible peaks were compared with the online database RRUFF (*93*).

### Leaching experiments

To test the leaching at various pH, we incubated a defined mass of glass sample of one grain size in 200 μl of water at 60°C in triplicate experiments. The water was adjusted to the desired pH with HCl/NaOH using a Thermo Fisher Scientific Orion 8220BNWP pH Electrode (Thermo Fisher Scientific, USA) to measure pH. Addition of carbonate (NaHCaCO_3_), citrate (C_6_H_5_Na_3_O_7_ 2H_2_O), ammonia (NH_4_Cl), and urea (CH_4_N_2_O), all from Sigma-Aldrich (USA) and Carl Roth GmBH (Germany), was done before pH adjustment. After 3 days, we recovered the samples and used 50 μl for measurement of ion composition in IC and 150 μl for either phosphate polymerization or nucleoside phosphorylation experiments.

### Ion chromatography

We used an IC system consisting of an autosampler (AS-DV, Thermo Fisher Scientific, USA) and two ICs, one for cation detection (Dionex Aquion, Thermo Fisher Scientific, USA) and one for anion detection (Dionex Integrion, Thermo Fisher Scientific, USA). For calibration, we used Dionex Seven Anion Standard II and Dionex Combined Six Cation Standard II from Thermo Fisher Scientific (USA) and prepared mixtures of polyphosphates (NaH_2_PO_4_, Na_3_P_3_O_9_, and Na_5_P_3_O_10_ from Sigma-Aldrich, USA, and Na_4_P_2_O_7_ from Carl Roth GmBH, Germany). The cation system consisted of an analytical column (Dionex IonPac CS12A), guard column (Dionex IonPac CG18), suppressor (Dionex CDRS 600), and conductivity detector (DS6 Heated Conductivity Cell), with a flow of 0.25 ml/min and 20 mM methanesulfonic acid (Carl Roth GmBH, Germany), 15-mA suppression, cell temperature of 40°C, and column temperature of 35°C. The anion system consisted of an analytical column (Dionex IonPac AS16 2 mm), guard column (Dionex IonPac AG16 2 mm), suppressor (Dionex ADRS 600 2 mm), eluent generator (EGC 500 KOH), trap column (Dionex CR-ATC 600), and conductivity detector (DS6 Heated Conductivity Cell). For separation, gradient elution starting with 57.5 mM KOH (for 10 min), a linear increase to 62.5 mM KOH over 2 min, isocratic elution with 62.5 mM KOH for 5 min, a direct step to 57.5 mM, and equilibration for 8 min was used with a flow of 0.25 ml/min and suppression at 47 mA, cell temperature at 40°C, and column temperature at 35°C. Data analysis was done using Chromeleon 7.2.10 (Thermo Fisher Scientific, USA).

### Polymerization of phosphate

For polymerization experiments, the samples remaining from the leaching experiments were separated into two fractions: 50 μl without P-glass and 100 μl with the P-glass. Both fractions were transferred to glass vials and heated in an oven (Memmert UNB 100 Oven, Germany) at 180°C without lids for 3 days. After that, samples were reeluted in IC water, and the supernatant was measured with IC. Experiments were done in triplicates.

### Phosphorylation experiments

For phosphorylation experiments, the remaining leachates were supplied with 0.5 mM adenosine (Sigma-Aldrich, USA). After that, samples were again separated into two fractions: 50 μl without P-glass and 100 μl with P-glass. The nonrock fraction was transferred to another vial, and both fractions were heated at 60°C with open lids over 3 days. Samples were then reeluted and analyzed using liquid chromatography–mass spectrometry (LC-MS). Experiments were done in triplicates.

### LC-MS measurements

Phosphorylation experiments were measured on an LC-MS system. The LC consisted of a Vanquish Flex (VF-S01-A), a binary pump (VF-P10-A-01), a heated column compartment (VH-C10-A), and a variable wavelength detector (VF-D40-A, all Thermo Fisher Scientific, USA), with column Symmetry C18 (3.5-μm pore size, 2.1-mm diameter, 150-mm length, and 100-Å particle size; WAT106005) (Waters, USA) and with eluents: A [LC-H_2_O with 0.1% (v/v) formic acid] and B [LC-acetonitrile with 0.1% (v/v) formic acid]. We applied a flow of 0.3 ml/min, column temperature of 30°C (still air), and detection of the ultraviolet (UV) absorption at 260 nm with 50 Hz. The elution protocol was started with 0% B, then an increase to 5% B over 12 min, a washing step at 40% B for 1 min, and equilibration at 0% B for 6 min. The LC was connected to a Q Exactive Plus Orbitrap HR/AM (Thermo Fisher Scientific, USA), with positive ionization and a resolution of 70,000, an automatic gain control target of 3 × 10^6^, and a maximum injection time of 200 ms. The heated electrospray ionization source had a sheath gas flow rate of 2, a spray voltage of 2.9 kV, a capillary temperature of 320°C, an auxiliary gas heater temperature of 50°C, and an S-lens radio frequency level of 50. For analysis, we extracted the main isotope mass ± 0.1 mass/charge ratio. Calibration was done using different levels of individual nucleotides and nucleoside, purchased from Sigma-Aldrich (USA).

### NMR spectroscopy

^1^H and ^31^P-NMR spectra were measured on a Bruker Avance III 500-MHz spectrometer equipped with a Prodigy BB cryoprobe at 298.15 K, with a ^1^H frequency of 500.13 MHz and a ^31^P frequency of 202.46 MHz. Quantitative ^31^P-NMR measurements were performed for ^31^P-NMR spectra. For ^31^P-NMR, a pulse sequence was set up with 128 transients (*ns* = 128), a P1 pulse length of 13.5 μs, which corresponds to ~90° pulse angle, and a d1 relaxation delay of 22.5 s to ensure full relaxation of nuclei between each transient. Relaxation delay d1 values were determined in a P-glass leachate sample, such that all ions and internal standards are fully quantitative (d1 ≥ 5× T1). Concentrations were determined in reference to a hexamethylphosphoramide (HMPA) internal standard, and yields of phosphorylated product were determined in reference to the total amount of leached phosphate (sum of the integral of all peaks in the ^31^P-NMR spectrum except the internal standard). A solvent suppression sequence was used for the measurement of ^1^H NMR spectra, as a 9:1 H_2_O:D_2_O solvent was used. All data were processed in MestReNova 14, and OriginPro 2020b.

### Imidazole phosphate formation in P-glass leachate

We immersed P-glass particles (200 mg/ml) in 1 M imidazole solution (pH 6.0; 9:1 H_2_O:D_2_O) with 1 mM HMPA internal standard for an initial leaching step of 2 days at 20°C under mild shaking in a thermoshaker. To initiate the reaction, solid potassium cyanate was added to a total concentration of 450 mM, and the formation of carbamoyl and imidazole phosphate was followed over time by ^31^P-NMR spectroscopy. For each NMR measurement, the P-glass suspension was centrifuged briefly, and the 500-μl supernatant was transferred to an NMR tube. After measurement, the solution was added back to the P-glass sample. Yields of leached orthophosphate [δ = 1.86 parts per million (ppm)], carbamoyl phosphate (δ = −2.30 ppm), and imidazole phosphate (δ = −4.73 ppm) were determined in reference to the HMPA internal standard (1 mM; δ = 29.65 ppm). Peak annotations are based on (*74*). Yields were fitted to a kinetic model as described below. A sample where the P-glass was removed by filtration with a spin filter after the initial leaching step but before addition of cyanate gave similar yields of carbamoyl and imidazole phosphate (fig. S15). All data points are shown in corresponding plots.

### Modeling of the formation of imidazole phosphate

To model the formation of imidazole phosphate, we used the chemical_kinetics (*94*) package in Python and defined the following set of equations with *p* (phosphate), *cy* (cyanate), *cp* (carbamoyl phosphate), *im* (imidazole) and *ip* (imidazole phosphate)

p+cy→cp (1)

cp+im→ip (2)

cp→p+waste (3)

ip→p+im (4)

cy→waste (5)

By fitting, we obtained the rate constants (shown in Fig. 3) *k_1_* = 0.00182 mM^−1^ hour^−1^, *k_2_* = 0.000012 mM^−1^ hour^−1^, *k_3_* = 0.4 hour^−1^, *k_4_* = 0.006 hour^−1^, and *k_5_* = 0.0147 hour^−1^.

### Glycerol phosphorylation by imidazole phosphate in a wet/dry cycle

A solution of 100 mM imidazole, 100 mM glycerol, and 100 mM potassium cyanate was adjusted to pH 7.3 using 5 M hydrochloric acid and subsequently added to P-glass (200 mg/ml). The suspension was left to react and accumulate imidazole phosphate for 24 hours while mixing on a rollerbank, after which it was pipetted onto a petri dish and left to dry with the lid off at 22°C for 24 hours, after which the petri dish was closed, sealed, and incubated at 50°C for 24 hours. To start the second cycle, the resulting paste was redissolved by addition of 100 mM potassium cyanate in 9:1 H_2_O:D_2_O, and the suspension of sample and P-glass was transferred back to an Eppendorf tube, after which the pH was adjusted back to 7.3 using 5 or 2 M hydrochloric acid. A 500-μl aliquot of the supernatant was taken for ^31^P-NMR measurements after brief centrifugation of the sample and was recombined with the sample after measurement. This procedure was repeated for 4 cycles, during which phosphorylated glycerol was accumulated. After the fourth cycle, the paste was dissolved in 9:1 H_2_O:D_2_O. Yields were determined in reference to the total amount of leached phosphate (sum of all peaks in the ^31^P-NMR spectrum): orthophosphate (δ = 2.10 ppm), carbamoyl phosphate (δ = −1.86 ppm), imidazole phosphate (δ = −4.53 ppm), glycerol 1-phosphate (δ = 4.01 ppm), and glycerol 2-phosphate (δ = 3.90 ppm, after ion exchange). The leached paramagnetic iron from the P-glass could reduce the signal-to-noise ratio in the ^31^P-NMR spectra due to line broadening. To make sure that we observed all the products formed in the reaction, we incubated 500 μl of the reaction supernatant after the fourth cycle with 200 mg of cation exchange resin (Chelex 100 resin sodium form 50 to 100 mesh) to replace the iron with sodium. After 40 min, the samples were filtered with a spin filter, and HMPA internal standard was added to a total concentration of 1 mM. ^31^P, ^1^H, and ^1^H–^31^P–heteronuclear multiple-bond correlation spectra were obtained.

## Supplementary Materials

### This PDF file includes:

Text S1 Figs. S1 to S21 Tables S1 to S3 References
