# Supplementary material for: Magmatic immiscibility provides phosphate for prebiotic chemistry
Source: Sci Adv. 2025 Nov 19;11(47):eadz2567. doi: 10.1126/sciadv.adz2567 (PMC12629179; doi:10.1126/sciadv.adz2567)
Supplement: Supplementary file 1 — Text S1 Figs. S1 to S21 Tables S1 to S3 References [file sciadv.adz2567_sm.pdf]

Science Advances
AAAS

Supplementary Materials for

Magmatic immiscibility provides phosphate for prebiotic chemistry

Daniel Weller *et al.*

Corresponding author: Daniel Weller, daniel.weller@min.uni-muenchen.de;
Thomas Matreux, thomas.matreux@espci.fr; Bettina Scheu, b.scheu@lmu.de

Sci. Adv. **11**, eadz2567 (2025)
DOI: 10.1126/sciadv.adz2567

This PDF file includes:

Text S1
Figs. S1 to S21
Tables S1 to S3
References

Text S1: Discussion of prebiotic relevance of reagents

The reagents used in the imidazole phosphate reaction all have prebiotically plausible synthesis pathways. Cyanate can be formed by photo-oxidation of hydrogen cyanide in the presence of a $[Fe(CN)_6]^{3-}$ or $[Cu(CN)_2]^-$ catalyst (*74*, *95*), catalysts that are readily formed by complexation of Fe^{2+} or Cu^+ with cyanide under prebiotically plausible conditions (*74*, *96–98*). This process captures solar energy to drive the imidazole phosphate reaction. In addition, cyanate concentrations of up to 12 mM were formed in spark discharge experiments with N_2, CO_2 and H_2 (*99*), showing that substantial amounts of cyanate can be accumulated for prebiotic chemistry. The production of imidazole phosphate further requires the presence of imidazole or other imidazolides (*73*). These are commonly used as catalysts and intermediates in prebiotic chemistry (*75*, *77*, *100*, *101*) and several prebiotic synthesis scenarios have been proposed. Imidazole can be synthesized prebiotically from formaldehyde, glyoxal and ammonia in aqueous solution (*76*), reagents that are believed to have been present on prebiotic Earth (*74*, *102*, *103*). It can also be synthesized by proton irradiation of a gaseous mixture of carbon monoxide and nitrogen over water, which produces several amino acids in the process (*104*).

Glycerol was used as an example substrate for phosphorylation by imidazole phosphate, which itself can phosphorylate a wide range of nucleophiles (*73*). It has several prebiotic synthesis routes, as reviewed by Gull and Pasek (*105*), including cyanosulfidic chemistry (*106*, *107*), the formose reaction (*108*), and interstellar ices (*109*, *110*). In addition, the detection of glycerol in the Murchison and Murray meteorites, carbonaceous chondrites, demonstrates that glycerol could have been delivered to Earth via meteorites (*111*, *112*).

Urea can, for example, be formed by the classical Wöhler reaction between ammonium and cyanate (*113*), or ammonium and cyanide (*114*) and via reaction of ammonia with carbamate, which is formed by hydrolysis of carbamoyl phosphate, as we use in Figure 3 (*115*). Urea is also formed as a byproduct in the cyanosulfidic scenario (*106*), and has been observed as a product in the spark discharge experiments by Miller (*116*). Urea has many uses in prebiotic chemistry, for example in ribonucleotide synthesis (*8*) and phosphorylation by (poly)phosphates as we show in this work (*7*, *8*, *117*). In addition, eutectic solvents consisting of, among others, urea have also been widely used in prebiotic chemistry (*118*, *119*).

Although a prebiotic synthesis of citrate is not yet known, citrate has been detected in the Murchison, Murray and Allan Hills (ALH) 83102 meteorites (*120*), indicating a plausible interstellar origin. In addition, other tricarboxylic acids, such as (iso)citroyl formate, aconitoyl formate, 3-carboxymalate and oxalomalate, have been shown to form in prebiotic analogues of the TCA cycle starting from pyruvate and glyoxylate (*121*, *122*). These alternative tricarboxylic acids can similarly chelate Ca^{2+} and have been shown to form coacervate protocells (*123*), similar to citrate.

Imidazole, glycerol, urea and citrate are all hydrolytically stable and could have therefore accumulated in a prebiotic environment.

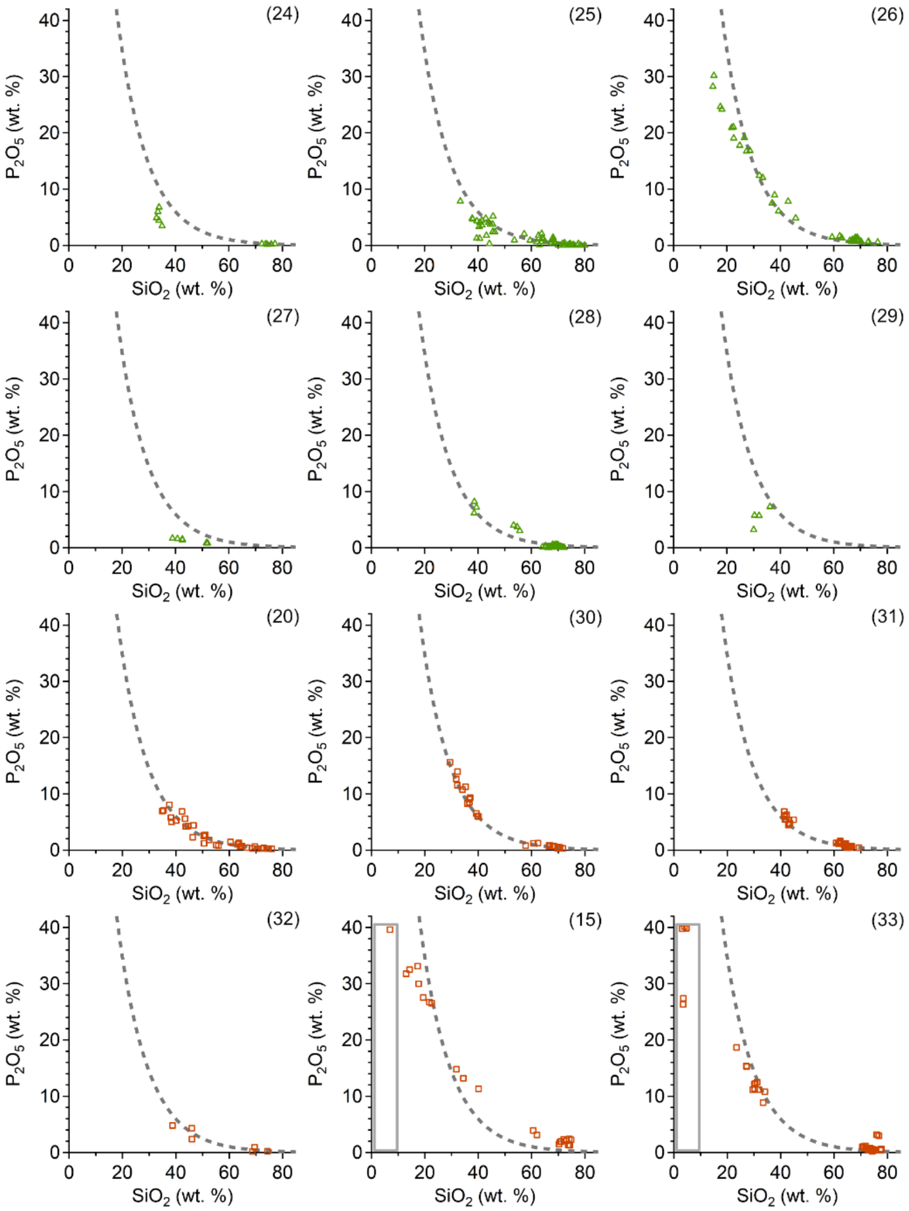

Figure S1: Phosphate enrichment in natural and synthetic liquid immiscible rock samples. *References from Fig. 2 are shown in detail, green data points indicate natural samples, orange show synthetic samples. The grey dashed line indicates the two-liquid immiscibility field following the model of Charlier and Grove* (*20*)*, yielding a fit of* $c_{P_2O_5} = 201.5 \cdot \exp(-0.088 \cdot c_{SiO_2})$ *with* $R^2 = 0.908$*. In* (*15*, *33*) *(bottom row), Fe-P-rich slags are shown in grey boxes (excluded from Fig. 2).*

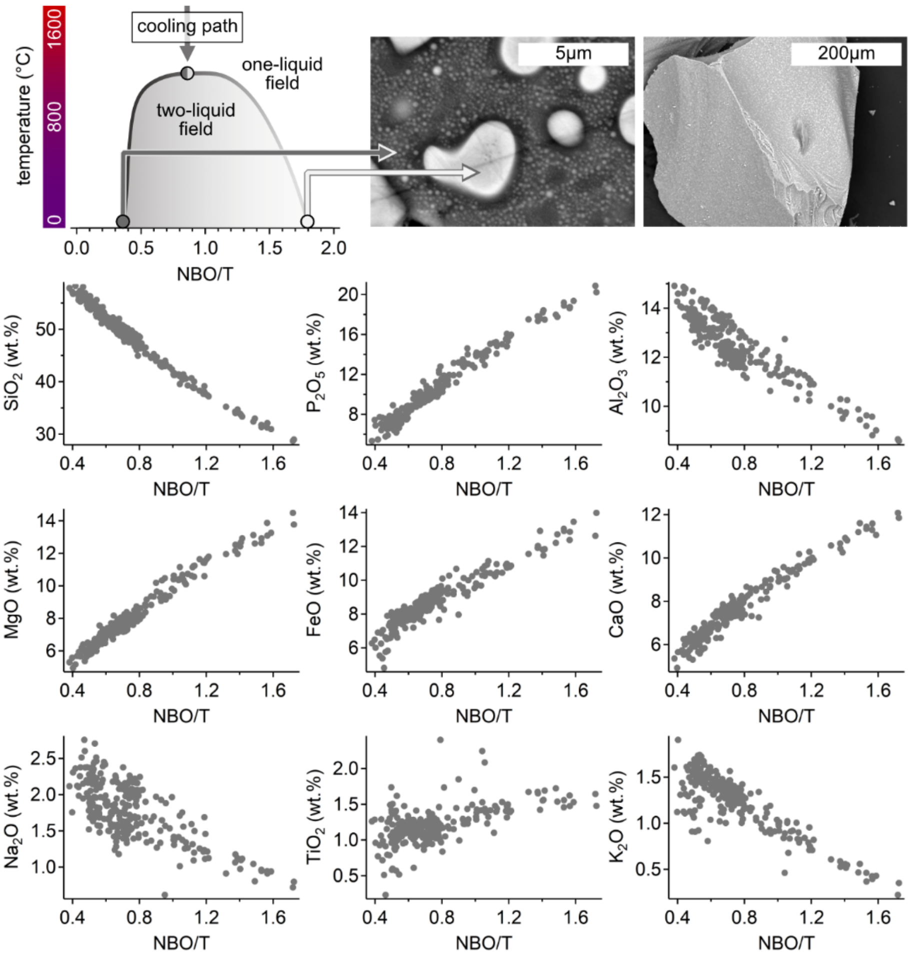

Figure S2: Immiscibility model from Charlier and Grove (20). *The non-bridging oxygens to tetrahedral cations (NBO/T) as a measure of polymerization is calculated according to Mysen et al. (61) and shown in the context of the petrological immiscibility from Charlier and Grove (20). On the right, SEM images of the unmixed glass show the Si-poor (high NBO/T) and Si-rich (low NBO/T) phases. In the lower part, the distributions of oxides present in the glass are shown over NBO/T, highlighting co-accumulation of elements. See also fig. S3 for their partition relative to* SiO_2.

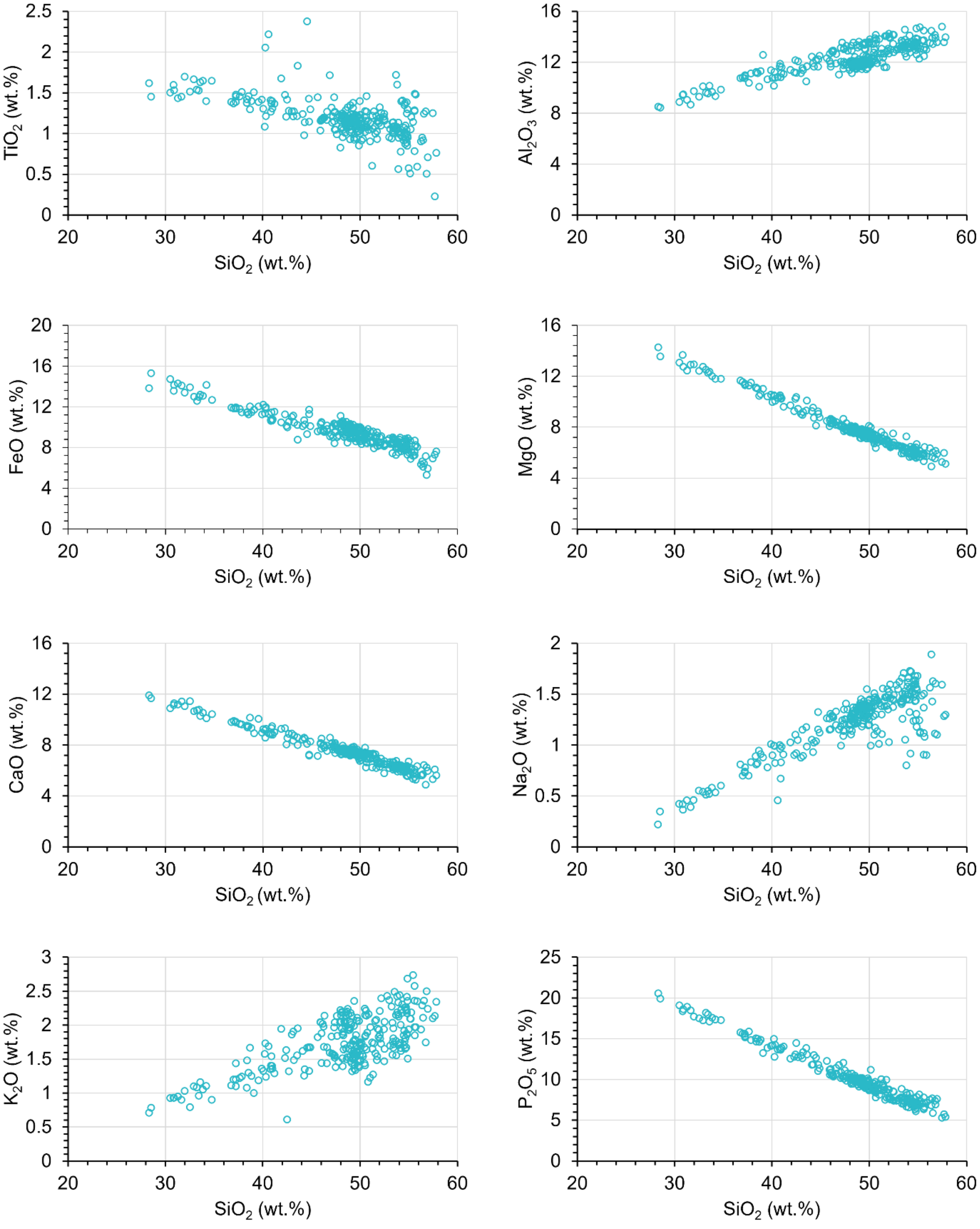

***Figure S3**: **Range of chemical composition of P-glass matrix and droplets measured with EPMA and SEM-BSE analysis**. All major elements are shown as a function of SiO_2. Al_2O_3, FeO, MgO, CaO, and P_2O_5 show the best correlation with SiO_2, while TiO_2 and alkali elements exhibit a wider scatter for measurements of the SiO_2-rich glass matrix.*

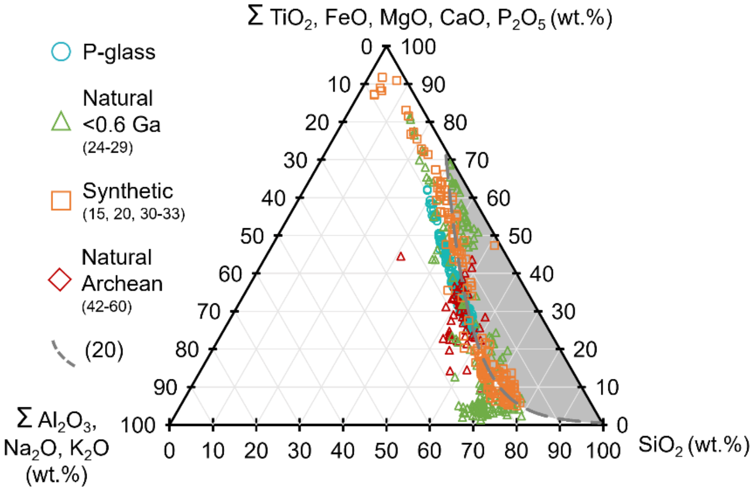

Figure S4: Ternary diagram of major elements. *Chemical characterization of P-glass (turquois circles) in comparison with liquid immiscible samples found in nature [green triangles,* (*24*–*29*)*], experimental samples synthesized in the laboratory [orange squares,* (*15*, *20*, *30*–*33*)*], and the composition of Archean rocks containing more than 0.5 wt % P_2O_5 [red triangles,* (*42*–*60*)*]. The two-liquid stability field (grey area) is based on the characterization of Charlier and Grove* (*20*)*, as shown in fig. S2. All datapoints are normalized to 100 wt % for this diagram. Two trends of natural and experimental samples are observable: One follows the outline of the two-liquid field of silicate melts, the other evolves from an average basaltic composition towards Fe-Ca-P slags and is affected by high water concentrations.*

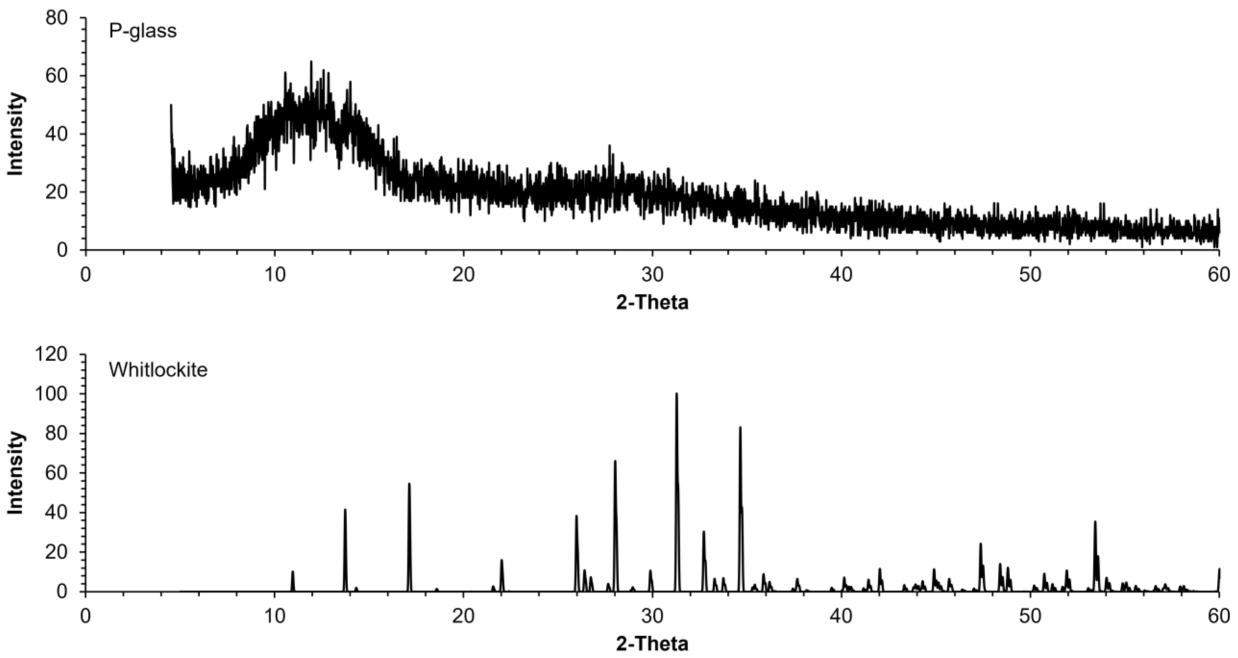

Figure S5: XRD analysis of P-enriched basaltic glass*. The absence of defined peaks indicates the absence of crystals in the glass. For reference, the spectrum of Whitlockite (RRUFF database ID: R070675) is shown.*

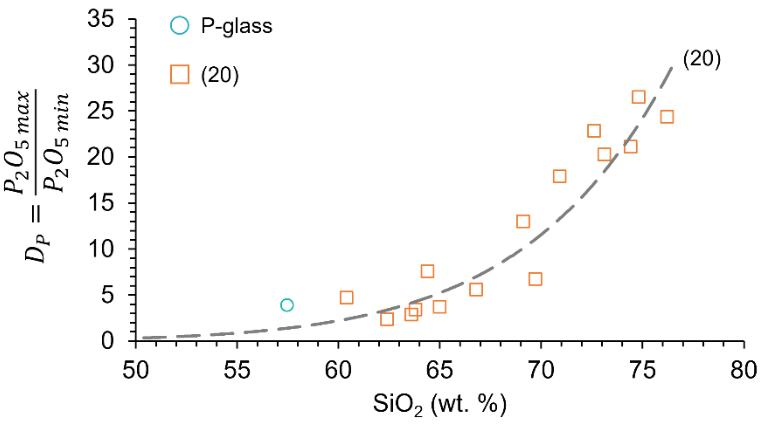

Figure S6: Concentration ratio of P-glass in the context of experiments by Charlier and Grove (20). *The concentration ratio of phosphorus is calculated from* $D_P = \frac{[P_2O_5]_{\text{max}}}{[P_2O_5]_{\text{min}}}$ *and shown over the* SiO_2 *concentration in the Si-rich glass phase. The concentration ratio of the P-glass (turquois circle) follows the experiments by Charlier and Grove (20) (orange squares). These data points yield a trend (grey line) with the equation* $D_P(c_{SiO_2}) = 2 \times 10^{-19} c_{SiO_2}{}^{10.712}$ *with* $R^2 = 0.8834$.

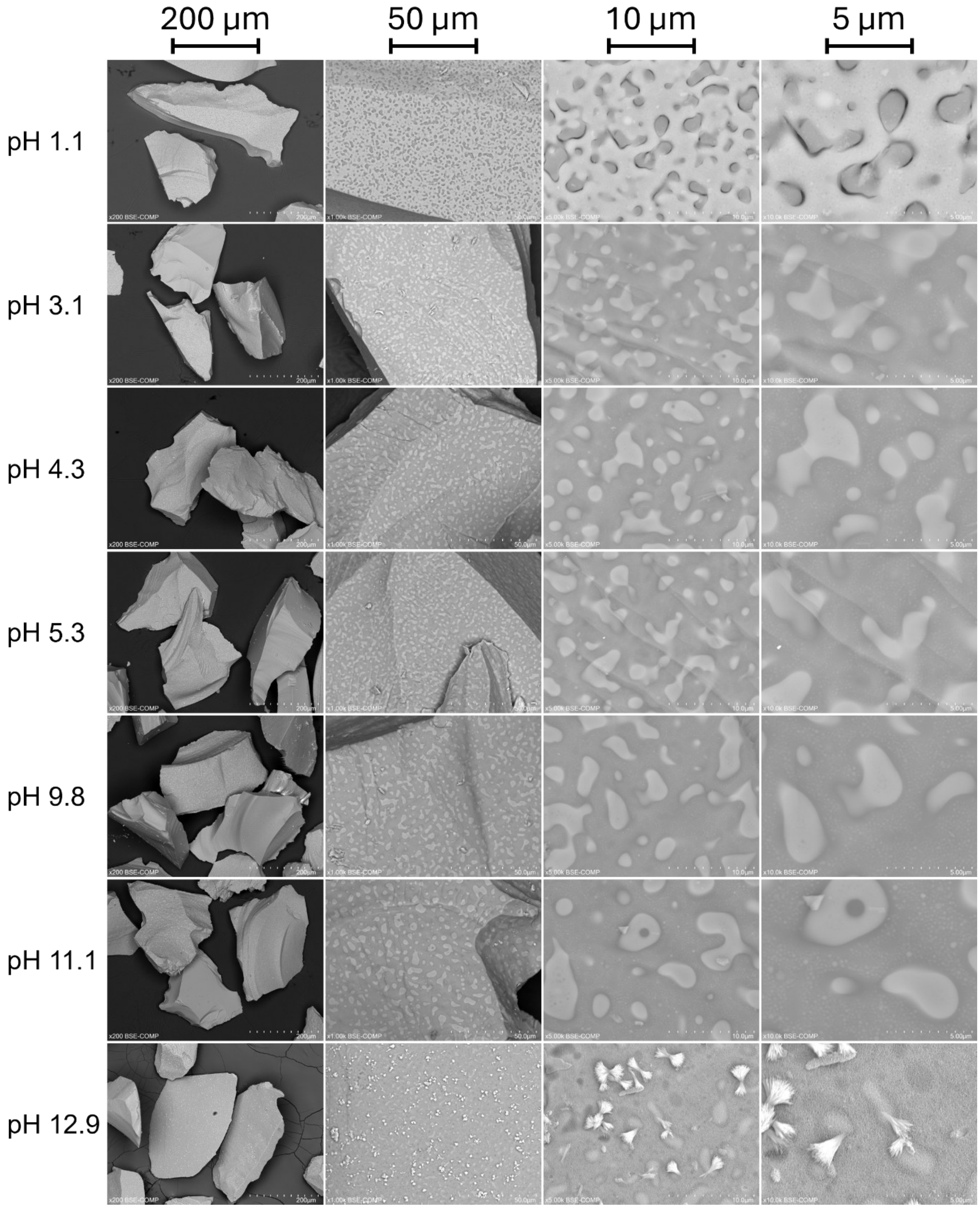

Figure S7: Back scatter electron imaging (BSE) of leached P-glass particles. *The two glassy phases show a pH dependent solubility. The P-enriched droplets show higher solubility in experiments performed at strongly acidic and basic pH values. For the latter, we observed precipitation of micrometer sized minerals. Images were taken with a scanning electron microscope (SEM) at magnifications 200x, 1000x, 5000x, and 10000x.*

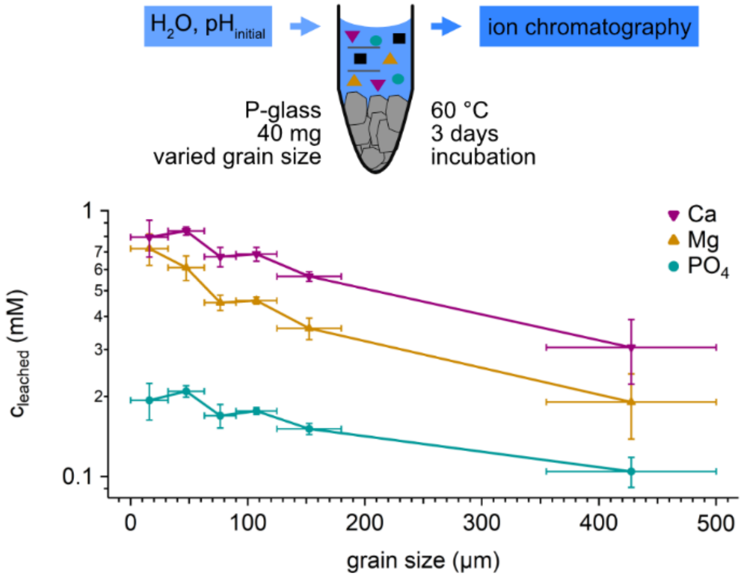

Figure S8: Variation of grain sizes for leaching. *Different grain size fractions of P-glass were incubated for 3 days at 60°C. Cation and phosphate concentrations evolve similarly over various grain sizes with absolute phosphate concentrations increasing towards smaller grain sizes. For the smallest grain size (<32 µm), wetting presumably becomes incomplete* (*63*)*. All error bars show the standard deviation (SD).*

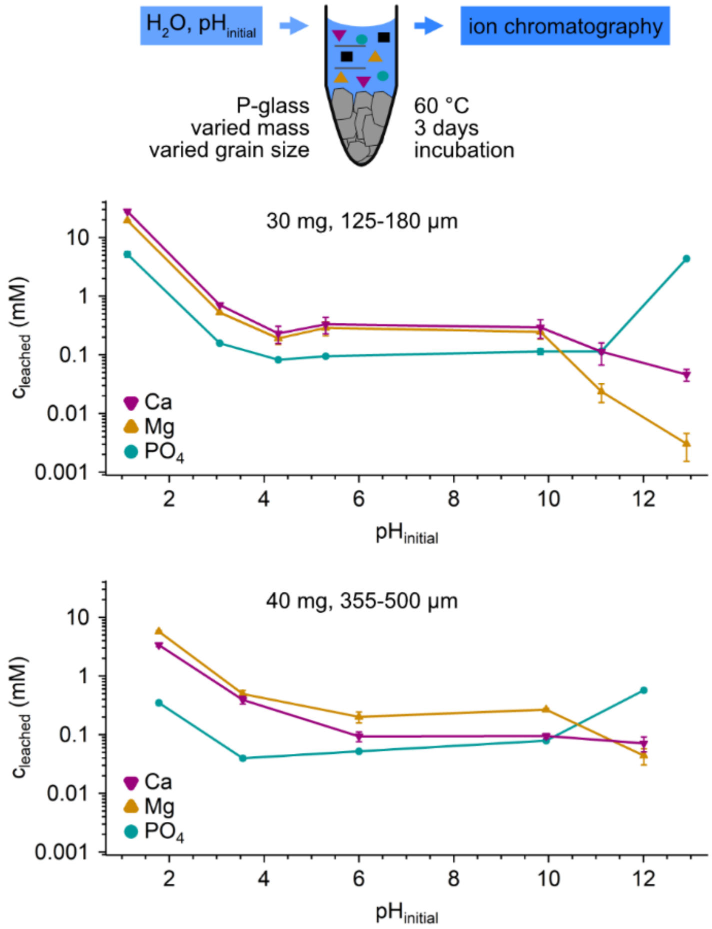

Figure S9: Detailed comparison of leaching from grain sizes 125-180 µm and 355-500 µm. *A more detailed comparison of ionic compositions of leachates for two grain sizes shows similar trends over pH. All error bars show the SD.*

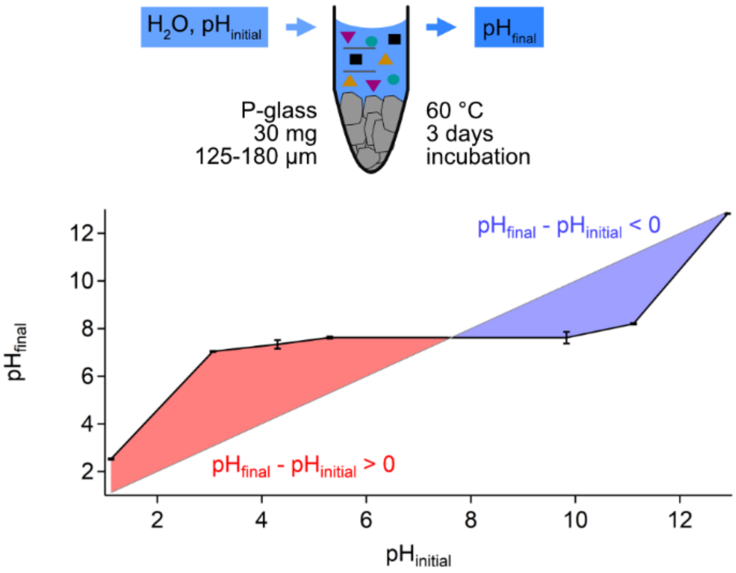

Figure S10: Evolution of pH during leaching. *Interaction with P-glass drives buffering towards neutral pH values which triggers partial reprecipitation (see main text and fig. S7). All error bars show the SD.*

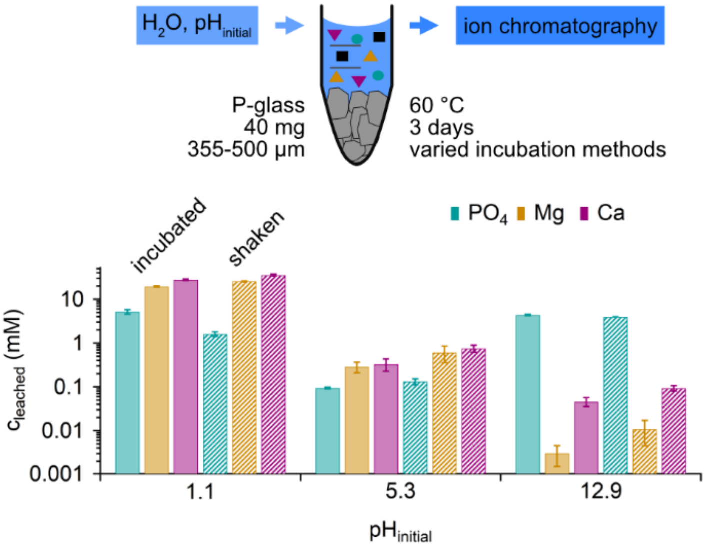

Figure S11: Leaching behavior for different incubation conditions. *Samples were either incubated without agitation or continuously agitated. Resulting leachate concentrations only showed minor differences. All error bars show the SD.*

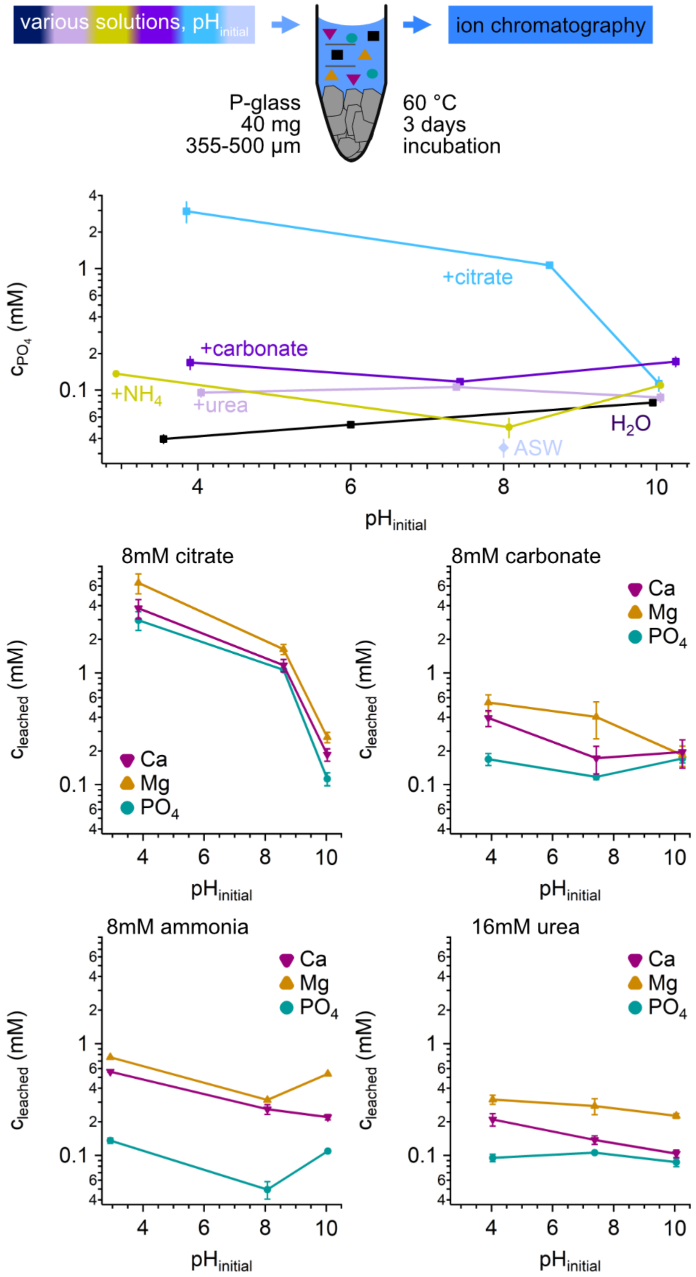

Figure S12: Leaching behavior for different solutions. *Samples were incubated in different prebiotically relevant chelation and condensing agents and artificial sea water (ASW) (63, 66). Leached concentrations are boosted in 8 mM citrate buffer while carbonate and urea buffers only weakly change the outcome compared to water (fig. S9). These additives were chosen as citrate (64) and carbonate (5) remove calcium as binding partner, while ammonia and urea facilitate phosphorylation (4, 7, 65, 124). All error bars show the SD.*

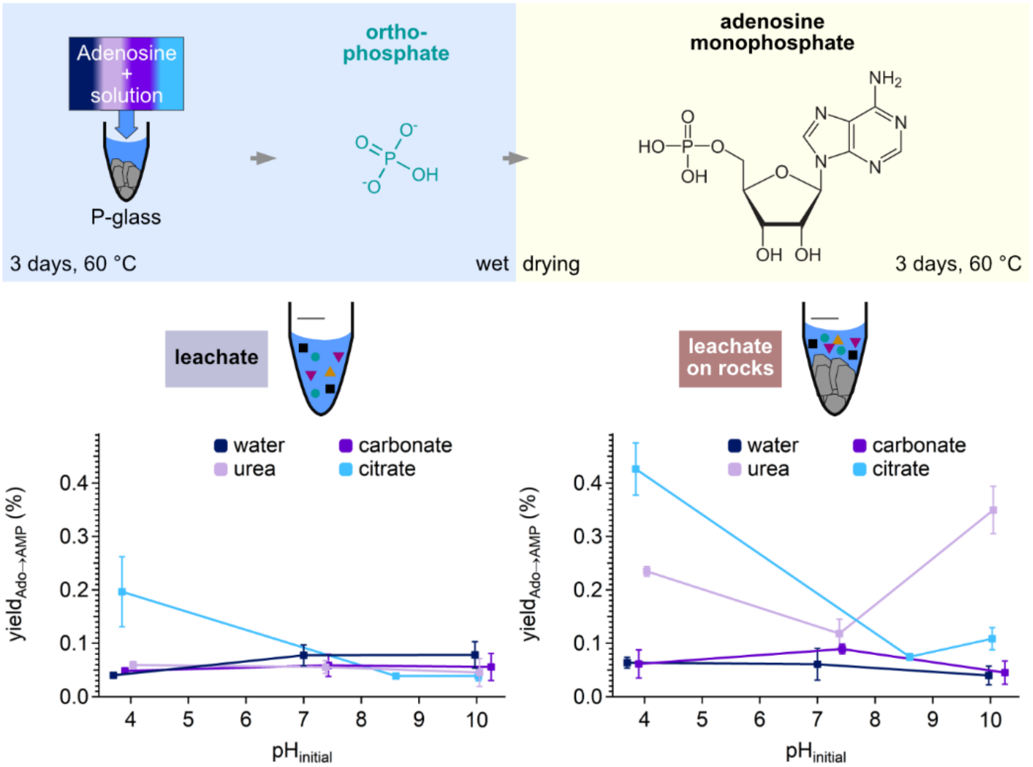

Figure S13: Phosphorylation of adenosine. *We studied the phosphorylation of adenosine, using various leachates from fig. S12. For this, we analyzed both a fraction heated without P-glass and on the leached P-glass. Presence of P-glass is overall positive for yields. Leaching and phosphorylation under addition of citrate shows best yields of AMP when starting from acidic conditions, presumably as citrate can chelate divalent cations such as calcium* (*64*)*. The presence of urea* (*4*, *7*, *65*, *124*) *increases yields for both acidic and alkaline conditions as condensing agent. All error bars show the SD.*

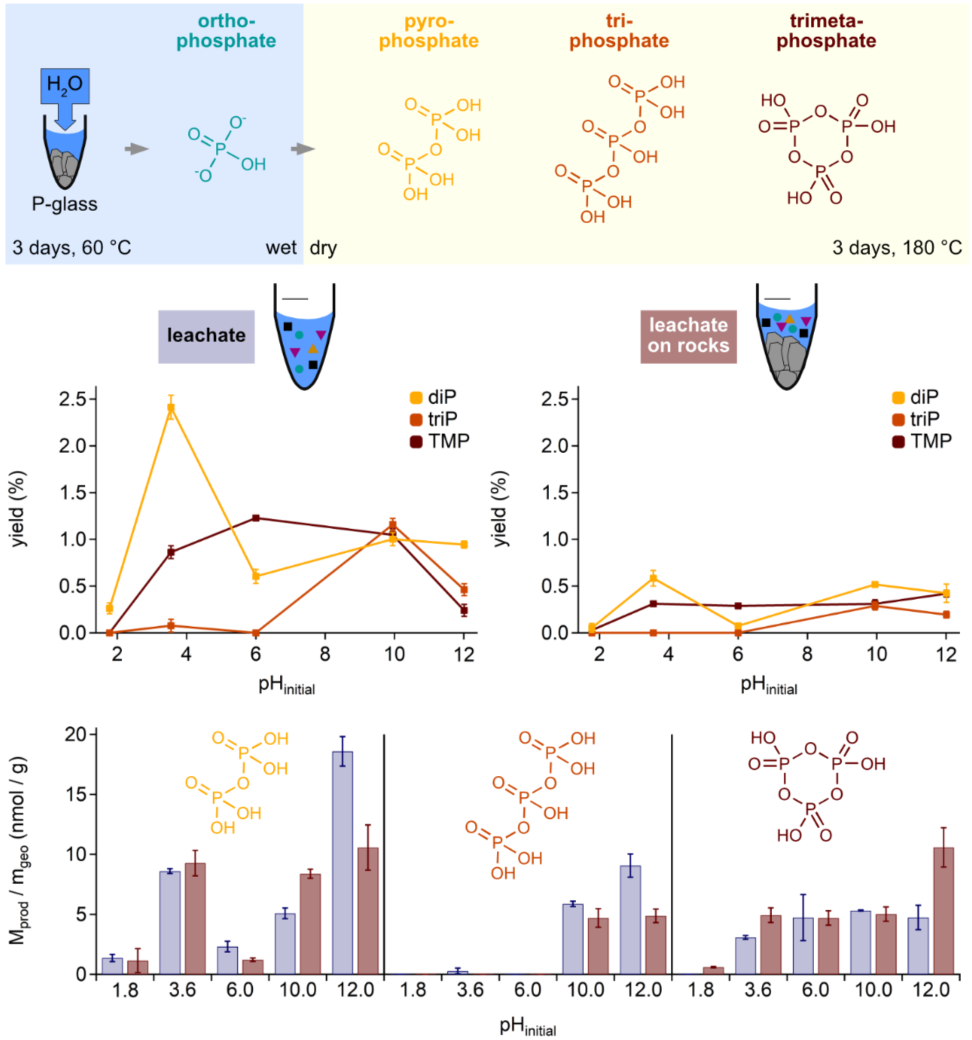

Figure S14: Polymerization of phosphates. *As orthophosphate exhibits poor reactivity, we investigated the potential of leached phosphate to polymerize into pyro-, tri-, and trimetaphosphate upon heating. Interestingly, concentration of redissolved products is lower in the case of reaction on P-glass, presumably due to adsorption. In the lower part, we display the quantity of synthesized polyphosphate per initial mass to avoid volume and mass dependent factors. All error bars show the SD.*

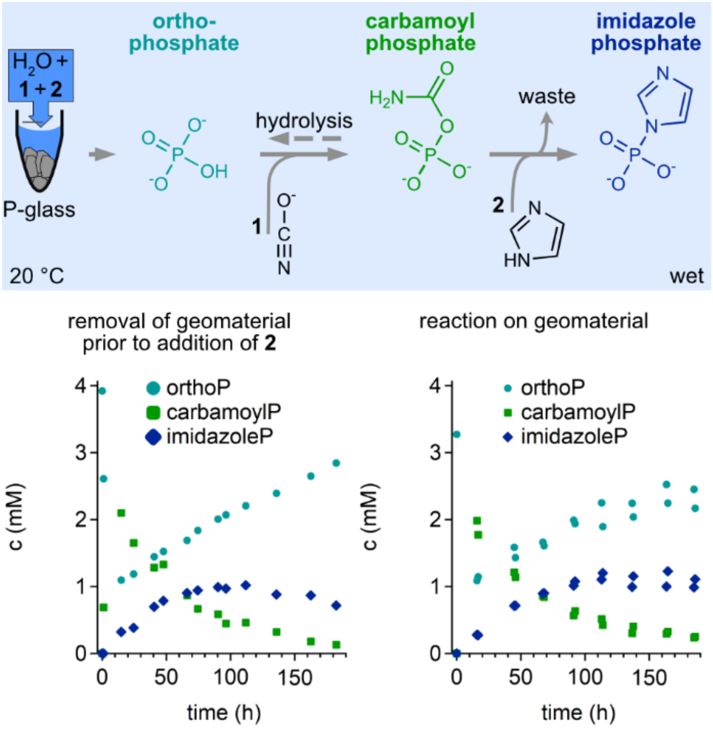

Figure S15: Formation of carbamoyl and imidazole phosphate. *Time evolution and yields are similar if the P-glass is removed prior to cyanate addition or if the reaction takes place in the presence of P-glass.*

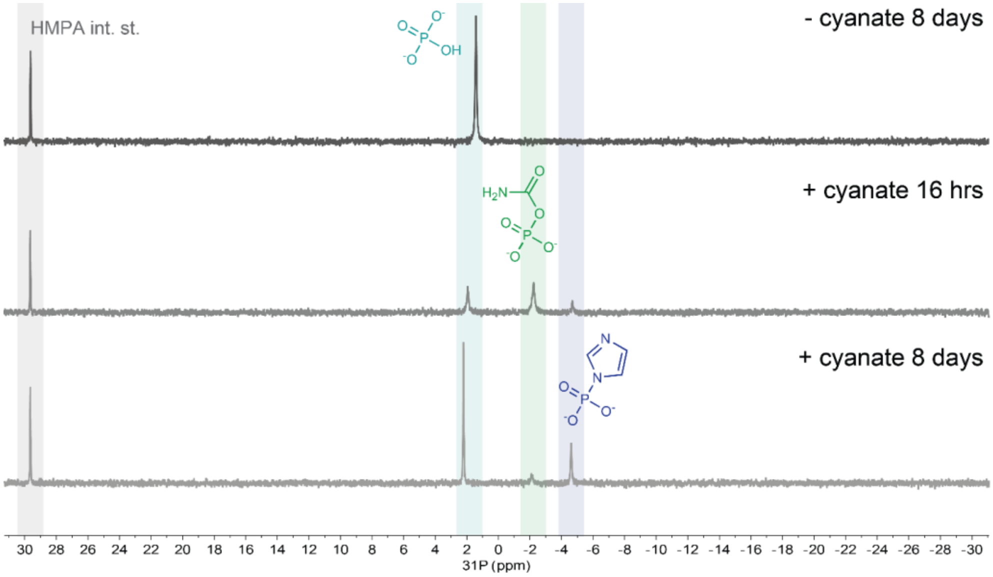

Figure S16: ^{31}P-NMR spectra of carbamoyl and imidazole phosphate formation. *Top: Spectrum of a sample without added cyanate, as control experiment, showing no conversion of orthophosphate. Middle: Sample with added cyanate, 16 hours after addition. Bottom: Sample with added cyanate, 7.7 days after addition. In all samples, the P-glass was present throughout the reaction.*

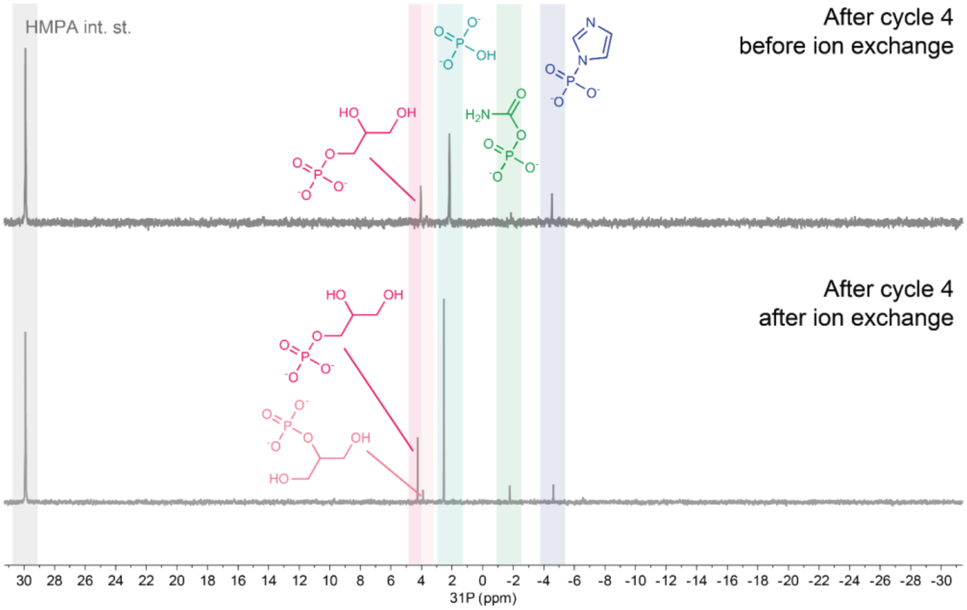

Figure S17: ^{31}P-NMR spectra of glycerol phosphorylation after the 4th wet/dry cycle before and after ion exchange. *Top: Before ion exchange, showing glycerol-1-phosphate as a product. Bottom: After ion exchange, in addition showing a peak for glycerol-2-phosphate.*

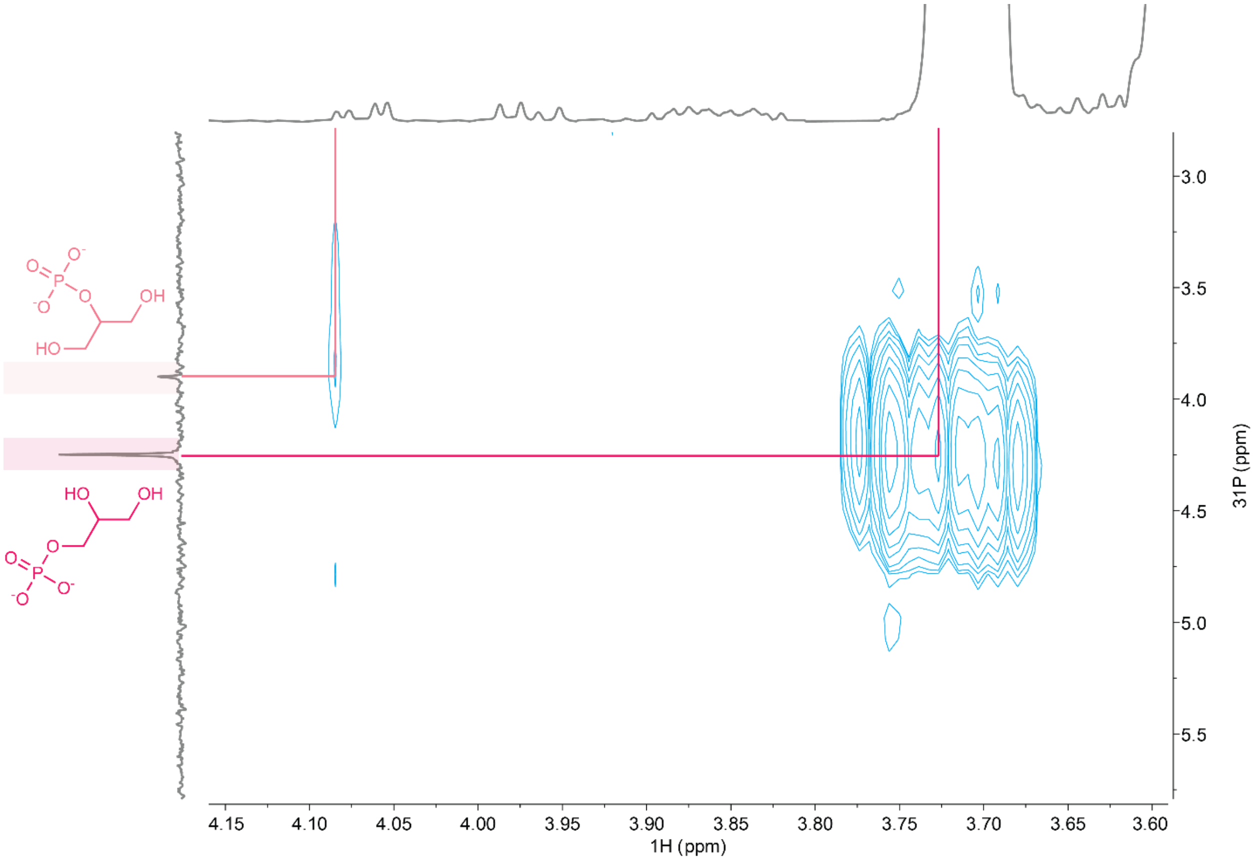

Figure S18: Detail of ^{1}H-^{31}P-HMBC NMR spectrum for the glycerol phosphorylation sample after ion exchange. *The peaks of glycerol-1-phosphate and glycerol-2-phosphate match those previously observed, see Maguire et al. Supplementary Figures 65 & 67* (*73*).

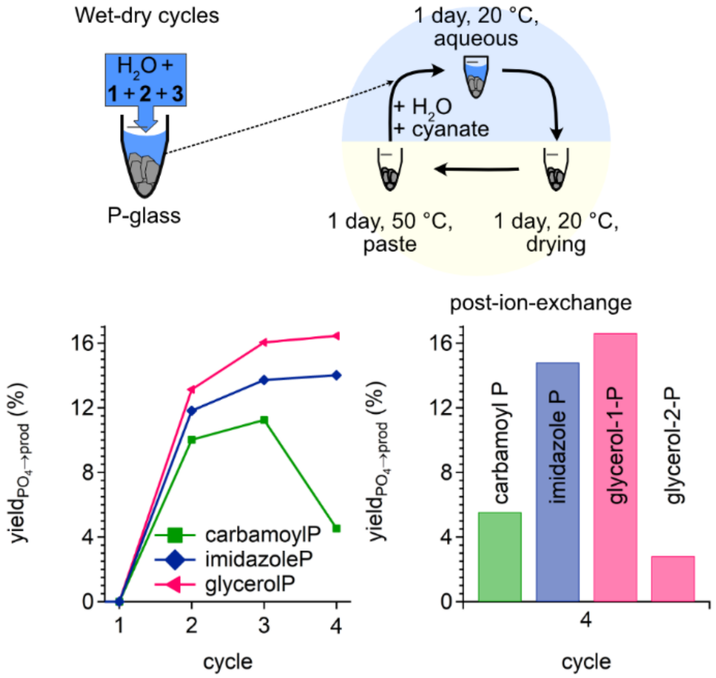

Figure S19: Composition of formed species after treatment with ion-exchange resin. *Samples were treated after a fourth cycle to analyze the effect of paramagnetic line broadening. The increased signal:noise ratio permitted detection of low quantities of formed glycerol-2-phosphate* (*73*).

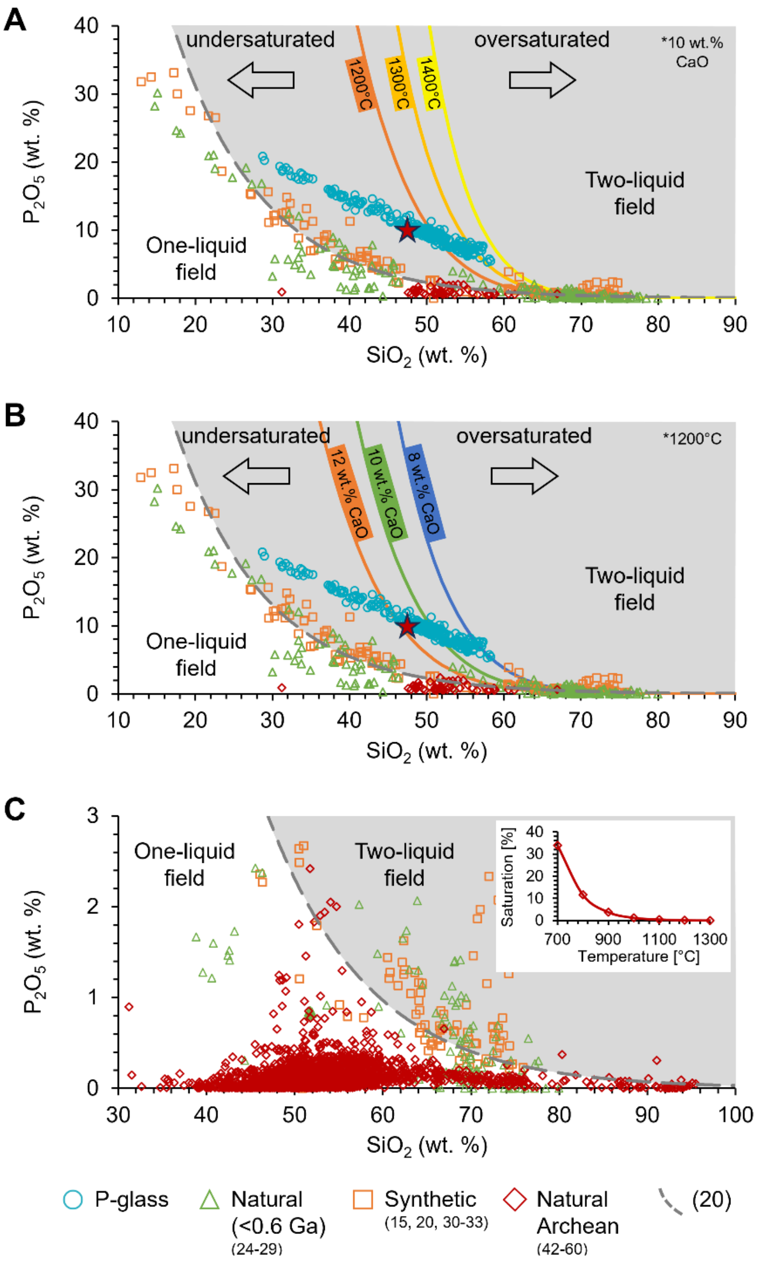

Figure S20: Saturation of phosphate minerals in immiscible and Archean rocks. (A) *The saturation of P_2O_5 in the melt phase decreases with higher SiO_2 concentration and lower temperature, leading to the formation of Apatite and Whitlockite. The colored lines, calculated after Tollari et al.* (*15*)*, show the SiO_2 dependent saturation curves at which the first phosphate minerals are formed. This is depicted for a fixed CaO concentration of 10 wt % and for different temperatures. Higher eruption temperatures of the magma in the Hadean could have resulted in the production of P-enriched glasses if cooling was quick enough. Immiscible melt droplets are P_2O_5-undersaturated for typical eruption temperatures >1000°C. Natural (green)* (*24*–*29*) *and synthetic (orange)* (*15*, *20*, *30*–*33*) *samples are shown for comparison.* ***(B)*** *CaO concentration has a similar effect on phosphate solubility as temperature. The more CaO in the melt, the higher the*

probability of apatite crystallization. Saturation curves are calculated for a fixed temperature of 1200°C. ***(C)*** *The phosphate concentration of Archean rocks* (*42–60*), *obtained from the GeoRoc database, described as basaltic volcanic rocks, are shown as a function of* SiO_2 *concentration. The inset shows the temperature dependent saturation of all Archean rocks. Above 1000°C, more than 98% of the samples are undersaturated with* P_2O_5*, meaning that apatite crystallization is suppressed, and that liquid immiscibility is possible.*

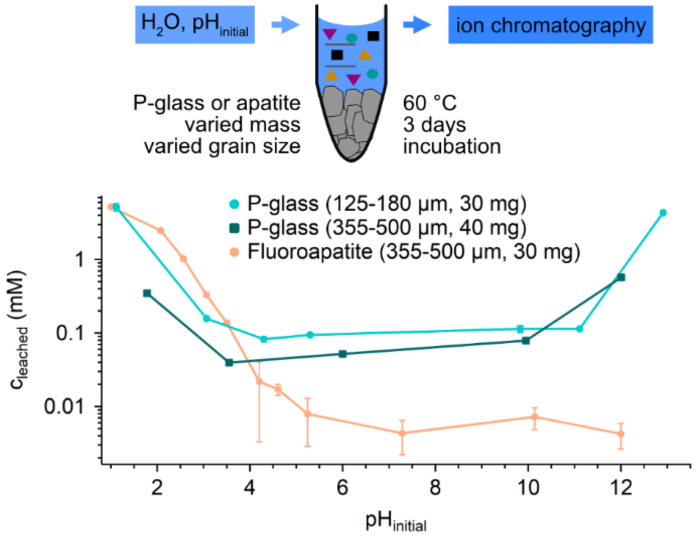

Figure S21: Comparison of leaching from P-glass to leaching from fluorapatite*. Leached concentrations were compared to prior results for natural fluorapatite* (*2*)*, showing strongly increased phosphate availability at neutral and alkaline pH values even though absolute phosphate content in the P-glass is four times lower. All error bars show the SD.*

**Oxide**	**Archean average** (*42–60*) **avg ± std**	**P-glass theoretical composition**
SiO_2	52.24 ± 4.32	52.54
TiO_2	1.46 ± 0.94	1.28
Al_2O_3	14.47 ± 3.05	13.28
FeO	11.81 ± 3.72	11.60
MgO	7.72 ± 5.75	9.10
CaO	8.24 ± 2.40	8.35
Na_2O	2.61 ± 1.66	2.43
K_2O	1.46 ± 1.30	1.43

Table S1. Normalized average Archean composition without phosphate compared to normalized P-glass theoretical composition without phosphate and volatiles. *To mimic an average Archean composition, we used the normalized average of oxides (without P_2O_5) and supplemented 10 wt % P_2O_5. All errors indicate the SD.*

Material	Unit	P-glass
SiO_2	g	47.49
TiO_2	g	1.15
Al_2O_3	g	12.00
Fe_2O_3	g	10.48
$CaCO_3$	g	13.46
MgO	g	8.22
Na_2CO_3	g	3.76
K_2CO_3	g	1.90
$(NH_4)_2HPO_4$	g	18.78
Sum	g	117.25

Table S2. Raw material weights for glass synthesis. *Oxides, carbonates, and diammonium hydrogen-phosphate were weighed with a high precision balance and homogenized before melting. Volatile exsolution of* CO_2*,* N_2*, and* H_2O *occurred during the process which is compensated by an initial mass > 100g.*

Oxide	P-glass	
	**Matrix**	**Droplets**
SiO_2	56.43 ± 0.98	30.93 ± 1.53
TiO_2	0.97 ± 0.24	1.55 ± 0.07
Al_2O_3	14.28 ± 0.40	9.17 ± 0.47
FeO	6.79 ± 0.76	14.03 ± 0.89
MgO	5.57 ± 0.37	13.08 ± 0.52
CaO	5.67 ± 0.38	11.27 ± 0.45
Na_2O	1.38 ± 0.24	0.42 ± 0.09
K_2O	2.18 ± 0.21	0.91 ± 0.11
P_2O_5	6.74 ± 0.78	18.65 ± 1.14

Table S3. Composition of synthesized P-glass measured by EPMA and SEM. *Measurements were done as described in the Methods. The average of ten spectra with lowest and highest* P_2O_5 *concentrations are calculated for the matrix and droplets respectively. All errors indicate the SD.*

REFERENCES AND NOTES

1. M. A. Pasek, Thermodynamics of prebiotic phosphorylation. *Chem. Rev.* **120**, 4690–4706 (2020).

2. T. Matreux, A. Schmid, M. Rappold, D. Weller, A. Z. Çalışkanoğlu, K. R. Moore, T. Bosak, D. B. Dingwell, K. Karaghiosoff, F. Guyot, B. Scheu, D. Braun, C. B. Mast, Heat flows solubilize apatite to boost phosphate availability for prebiotic chemistry. *Nat. Commun.* **16**, 1809 (2025).

3. C. Chaïrat, J. Schott, E. H. Oelkers, J.-E. Lartigue, N. Harouiya, Kinetics and mechanism of natural fluorapatite dissolution at 25°C and pH from 3 to 12. *Geochim. Cosmochim. Acta* **71**, 5901–5912 (2007).

4. A. W. Schwartz, Prebiotic phosphorylation-nucleotide synthesis with apatite. *Biochim. Biophys. Acta BBA Nucleic Acids Protein Synth.* **281**, 477–480 (1972).

5. J. D. Toner, D. C. Catling, A carbonate-rich lake solution to the phosphate problem of the origin of life. *Proc. Natl. Acad. Sci. U.S.A.* **117**, 883–888 (2020).

6. M. Gull, M. Pasek, Is struvite a prebiotic mineral? *Life* **3**, 321–330 (2013).

7. R. Lohrmann, L. E. Orgel, Prebiotic synthesis: Phosphorylation in aqueous solution. *Science* **161**, 64–66 (1968).

8. M. W. Powner, B. Gerland, J. D. Sutherland, Synthesis of activated pyrimidine ribonucleotides in prebiotically plausible conditions. *Nature* **459**, 239–242 (2009).

9. B. L. Hess, S. Piazolo, J. Harvey, Lightning strikes as a major facilitator of prebiotic phosphorus reduction on early Earth. *Nat. Commun.* **12**, 1535 (2021).

10. B. Herschy, S. J. Chang, R. Blake, A. Lepland, H. Abbott-Lyon, J. Sampson, Z. Atlas, T. P. Kee, M. A. Pasek, Archean phosphorus liberation induced by iron redox geochemistry. *Nat. Commun.* **9**, 1346 (2018).

11. M. A. Pasek, D. S. Lauretta, Aqueous corrosion of phosphide minerals from iron meteorites: A highly reactive source of prebiotic phosphorus on the surface of the early Earth. *Astrobiology* **5**, 515–535 (2005).

12. A. E. Beswick, I. S. E. Carmichael, Constraints on mantle source compositions imposed by phosphorus and the rare-earth elements. *Contrib. Mineral. Petrol.* **67**, 317–330 (1978).

13. C. Ma, Y. Tang, J. Ying, H. Zhang, The phosphorus budget of the silicate Earth based on an updated estimate of the P/Nd ratio. *J. Geophys. Res. Solid Earth* **128**, e2022JB025384 (2023).

14. M. J. Toplis, G. Libourel, M. R. Carroll, The role of phosphorus in crystallisation processes of basalt: An experimental study. *Geochim. Cosmochim. Acta* **58**, 797–810 (1994).

15. N. Tollari, M. J. Toplis, S.-J. Barnes, Predicting phosphate saturation in silicate magmas: An experimental study of the effects of melt composition and temperature. *Geochim. Cosmochim. Acta* **70**, 1518–1536 (2006).

16. E. B. Watson, Apatite and phosphorus in mantle source regions: An experimental study of apatite/melt equilibria at pressures to 25 kbar. *Earth Planet. Sci. Lett.* **51**, 322–335 (1980).

17. A. Höskuldsson, R. S. J. Sparks, M. R. Carroll, Constraints on the dynamics of subglacial basalt eruptions from geological and geochemical observations at Kverkfjöll, NE-Iceland. *Bull. Volcanol.* **68**, 689–701 (2006).

18. F. J. Ryerson, P. C. Hess, Implications of liquid-liquid distribution coefficients to mineral-liquid partitioning. *Geochim. Cosmochim. Acta* **42**, 921–932 (1978).

19. E. Roedder, P. W. Weiblen, Lunar petrology of silicate melt inclusions, Apollo 11 rocks. *Geochim. Cosmochim. Acta Suppl.* **1**, 801 (1970).

20. B. Charlier, T. L. Grove, Experiments on liquid immiscibility along tholeiitic liquid lines of descent. *Contrib. Mineral. Petrol.* **164**, 27–44 (2012).

21. M. Bogaerts, M. W. Schmidt, Experiments on silicate melt immiscibility in the system Fe_2SiO_4–$KAlSi_3O_8$–SiO_2–CaO–MgO–TiO_2–P_2O_5 and implications for natural magmas. *Contrib. Mineral. Petrol.* **152**, 257–274 (2006).

22. D. M. Collins, N. D'Souza, C. Panwisawas, C. Papadaki, G. D. West, A. Kostka, P. Kontis, Spinodal decomposition versus classical γ′ nucleation in a nickel-base superalloy powder: An in-situ neutron diffraction and atomic-scale analysis. *Acta Mater.* **200**, 959–970 (2020).

23. I. B. A. Smokers, B. S. Visser, W. P. Lipiński, K. K. Nakashima, E. Spruijt, Phase-separated droplets can direct the kinetics of chemical reactions including polymerization, self-replication and oscillating networks. *ChemSystemsChem* **7**, e202400056 (2025).

24. B. Charlier, O. Namur, T. L. Grove, Compositional and kinetic controls on liquid immiscibility in ferrobasalt–rhyolite volcanic and plutonic series. *Geochim. Cosmochim. Acta* **113**, 79–93 (2013).

25. A. R. Philpotts, Compositions of immiscible liquids in volcanic rocks. *Contrib. Mineral. Petrol.* **80**, 201–218 (1982).

26. V. S. Kamenetsky, B. Charlier, L. Zhitova, V. Sharygin, P. Davidson, S. Feig, Magma chamber–scale liquid immiscibility in the Siberian Traps represented by melt pools in native iron. *Geology* **41**, 1091–1094 (2013).

27. V. C. Honour, M. B. Holness, B. Charlier, S. C. Piazolo, O. Namur, T. J. Prosa, I. Martin, R. T. Helz, J. Maclennan, M. M. Jean, Compositional boundary layers trigger liquid unmixing in a basaltic crystal mush. *Nat. Commun.* **10**, 4821 (2019).

28. B. Charlier, O. Namur, M. J. Toplis, P. Schiano, N. Cluzel, M. D. Higgins, J. V. Auwera, Large-scale silicate liquid immiscibility during differentiation of tholeiitic basalt to granite and the origin of the Daly gap. *Geology* **39**, 907–910 (2011).

29. L.-C. Kuo, J. H. Lee, E. J. Essene, D. R. Peacor, Occurrence, chemistry, and origin of immiscible silicate glasses in a tholeiitic basalt: A TEM/AEM study. *Contrib. Mineral. Petrol.* **94**, 90–98 (1986).

30. V. C. Honour, M. B. Holness, J. L. Partridge, B. Charlier, Microstructural evolution of silicate immiscible liquids in ferrobasalts. *Contrib. Mineral. Petrol.* **174**, 77 (2019).

31. L. M. Lino, P. R. Carvalho, S. R. F. Vlach, F. R. Quiroz-Valle, Evidence for silicate liquid immiscibility in recharging, alkali-rich tholeiitic systems: The role of unmixing in the petrogenesis of intermediate, layered plutonic bodies and bimodal volcanic suites. *Lithos* **450-451**, 107193 (2023).

32. P. C. Hess, M. J. Rutherford, R. N. Guillemette, F. J. Ryerson, H. A. Tuchfeld, Residual products of fractional crystallization of lunar magmas. *Proc. Lunar Sci. Conf. 6th* (1975), pp. 895–909.

33. T. Hou, B. Charlier, F. Holtz, I. Veksler, Z. Zhang, R. Thomas, O. Namur, Immiscible hydrous Fe–Ca–P melt and the origin of iron oxide-apatite ore deposits. *Nat. Commun.* **9**, 1415 (2018).

34. A. Soldati, J. A. Farrell, R. Wysocki, J. A. Karson, Imagining and constraining ferrovolcanic eruptions and landscapes through large-scale experiments. *Nat. Commun.* **12**, 1711 (2021).

35. M. J. Toplis, D. B. Dingwell, G. Libourel, The effect of phosphorus on the iron redox ratio, viscosity, and density of an evolved ferro-basalt. *Contrib. Mineral. Petrol.* **117**, 293–304 (1994).

36. A. R. McBirney, The Skaergaard Layered Series: I. Structure and average compositions. *J. Petrol.* **30**, 363–397 (1989).

37. A. R. McBirney, R. M. Noyes, Crystallization and layering of the Skaergaard intrusion. *J. Petrol.* **20**, 487–554 (1979).

38. C. Manikyamba, S. M. Naqvi, Late archaean mantle fertility: Constraints from metavolcanics of the Sandur Schist Belt, India. *Gondwana Res.* **1**, 69–89 (1997).

39. T. Keller, F. Tornos, J. M. Hanchar, D. K. Pietruszka, A. Soldati, D. B. Dingwell, J. Suckale, Genetic model of the El Laco magnetite-apatite deposits by extrusion of iron-rich melt. *Nat. Commun.* **13**, 6114 (2022).

40. J. O. Nyström, F. Henríquez, J. A. Naranjo, H. R. Naslund, Magnetite spherules in pyroclastic iron ore at El Laco, Chile. *Am. Mineral.* **101**, 587–595 (2016).

41. K. Chen, R. L. Rudnick, Z. Wang, M. Tang, R. M. Gaschnig, Z. Zou, T. He, Z. Hu, Y. Liu, How mafic was the Archean upper continental crust? Insights from Cu and Ag in ancient glacial diamictites. *Geochim. Cosmochim. Acta* **278**, 16–29 (2020).

42. S. M. Hussain, S. M. Naqvi, T. Gnaneshwar Rao, Geochemistry and significance of mafic-ultramafic rocks from the southern part of the Holenarasipur Schist Belt, Karnataka. *J. Geol. Soc. India* **23**, 19–31 (1982).

43. H. Papunen, T. Halkoaho, E. J. Luukkonen, E. Luukkonen, *Archaean Evolution of the Tipasjärvi-Kuhmo-Suomussalmi Greenstone Complex*, *Finland* (Geological Survey of Finland, 2009).

44. L. P. Mare, C. J. S. Fourie, New geochemical and palaeomagnetic results from neoarchean dyke swarms in the Badplaas-Barberton area, South Africa. *South Afr. J. Geol.* **115**, 145–170 (2012).

45. A. Riganti, A. H. Wilson, Geochemistry of the mafic/ultramafic volcanic associations of the Nondweni greenstone belt, South Africa, and constraints on their petrogenesis. *Lithos* **34**, 235–252 (1995).

46. P. Hollings, R. Kerrich, An Archean arc basalt–Nb-enriched basalt–adakite association: The 2.7 Ga Confederation assemblage of the Birch–Uchi greenstone belt, Superior Province. *Contrib. Mineral. Petrol.* **139**, 208–226 (2000).

47. R. Kerrich, A. Polat, D. Wyman, P. Hollings, Trace element systematics of Mg-, to Fe-tholeiitic basalt suites of the Superior Province: Implications for Archean mantle reservoirs and greenstone belt genesis. *Lithos* **46**, 163–187 (1999).

48. T. Wu, A. Polat, R. Frei, B. J. Fryer, K.-G. Yang, T. Kusky, Geochemistry, Nd, Pb and Sr isotope systematics, and U–Pb zircon ages of the Neoarchean Bad Vermilion Lake greenstone

belt and spatially associated granitic rocks, western Superior Province, Canada. *Precambrian Res.* **282**, 21–51 (2016).

49. P. Liou, H. Shan, F. Liu, J. Guo, Petrogenesis of Neoarchean metavolcanic rocks in Changyukou, Northwestern Hebei: Implications for the transition stage from a compressional to an extensional regime for the North China Craton. *Lithos* **274-275**, 53–72 (2017).

50. R. P. Hartlaub, L. M. Heaman, K. E. Ashton, T. Chacko, The Archean Murmac Bay Group: Evidence for a giant Archean rift in the Rae Province. *Canada. Precambrian Res.* **131**, 345–372 (2004).

51. J. C. Ordóñez-Calderón, A. Polat, B. J. Fryer, J. E. Gagnon, J. G. Raith, P. W. U. Appel, Evidence for HFSE and REE mobility during calc-silicate metasomatism, Mesoarchean (□3075Ma) Ivisaartoq greenstone belt, southern West Greenland. *Precambrian Res.* **161**, 317–340 (2008).

52. A. A. Garde, Accretion and evolution of an Archaean high-grade grey gneiss – amphibolite complex: The Fiskefjord area, southern West Greenland. *Geol. Greenl. Surv. Bull.* **177**, 1–115 (1997).

53. C. Manikyamba, R. Kerrich, Geochemistry of alkaline basalts and associated high-Mg basalts from the 2.7Ga Penakacherla Terrane, Dharwar craton, India: An Archean depleted mantle-OIB array. *Precambrian Res.* **188**, 104–122 (2011).

54. S. N. Charan, S. M. Naqvi, S. L. Ramesh, Geology and geochemistry of spinifex-textured peridotitic komatiite from Mayasandra Schist Belt, Karnataka. *J. Geol. Soc. India* **32**, 343–350 (1988).

55. N. Arndt, G. Bruzak, T. Reischmann, “The oldest continental and oceanic plateaus: Geochemistry of basalts and komatiites of the Pilbara Craton, Australia,” in *Mantle Plumes: Their Identification through Time* (Geological Society of America, 2001); https://pubs.geoscienceworld.org/books/book/518/chapter/3801285.

56. D. T. Murphy, D. Wiemer, V. C. Bennett, T. Spring, J. Trofimovs, H. E. Cathey, Paleoarchean variole-bearing metabasalts from the East Pilbara Terrane formed by hydrous fluid phase exsolution and implications for Archean greenstone belt magmatic processes. *Precambrian Res.* **357**, 106114 (2021).

57. N. Thébaud, S. Barnes, M. Fiorentini, Komatiites of the Wildara-Leonora Belt, Yilgarn Craton, WA: The missing link in the Kalgoorlie Terrane? *Precambrian Res.* **196-197**, 234–246 (2012).

58. D. Saha, P. Bachhar, G. K. Deb, S. Patranabis-Deb, A. Banerjee, Tectonic evolution of the Paleoarchean to Mesoarchean Badampahar-Gorumahisani belt, Singhbhum craton, India – Implications for coexisting arc and plume signatures in a granite-greenstone terrain. *Precambrian Res.* **357**, 106094 (2021).

59. S. Hussain, S. Naqvi, Geological, geophysical and geochemical studies over the Holenarasipur schist belt, Dharwar Craton, India. *Precambrian South India Mem. Geol. Soc. India* **4**, 73–95 (1983).

60. P. C. C. D. Costa, V. A. V. Girardi, Petrology, geochemistry and Sr-Nd isotopes of the paleoproterozoic mafic dykes from the Goiás-Crixás Archean Block, Goiás State, Brazil. *Rev. Bras. Geociências* **35**, 135–150 (2005).

61. B. O. Mysen, D. Virgo, F. A. Seifert, Relationships between properties and structure of aluminosilicate melts. *Am. Mineral.* **70**, 88–105 (1985).

62. T. Matreux, B. Altaner, J. Raith, D. Braun, C. B. Mast, U. Gerland, Formation mechanism of thermally controlled pH gradients. *Commun. Phys.* **6**, 14 (2023).

63. T. Matreux, K. Le Vay, A. Schmid, P. Aikkila, L. Belohlavek, A. Z. Çalışkanoğlu, E. Salibi, A. Kühnlein, C. Springsklee, B. Scheu, D. B. Dingwell, D. Braun, H. Mutschler, C. B. Mast, Heat flows in rock cracks naturally optimize salt compositions for ribozymes. *Nat. Chem.* **13**, 1038–1045 (2021).

64. D. N. Misra, Interaction of citric acid with hydroxyapatite: Surface exchange of ions and precipitation of calcium citrate. *J. Dent. Res.* **75**, 1418–1425 (1996).

65. M. Gull, A. Omran, T. Feng, M. A. Pasek, Silicate-, magnesium ion-, and urea-induced prebiotic phosphorylation of uridine via pyrophosphate; Revisiting the hot drying water pool scenario. *Life* **10**, 122 (2020).

66. T. M. McCollom, C. Donaldson, Experimental constraints on abiotic formation of tubules and other proposed biological structures in subsurface volcanic glass. *Astrobiology* **19**, 53–63 (2019).

67. D. Gan, J. Ying, Y. Zhao, Prebiotic chemistry: The role of trimetaphosphate in prebiotic chemical evolution. *Front. Chem.* **10**, 941228 (2022).

68. T. Matreux, P. Aikkila, B. Scheu, D. Braun, C. B. Mast, Heat flows enrich prebiotic building blocks and enhance their reactivity. *Nature* **628**, 110–116 (2024).

69. H. Boigenzahn, P. Gagrani, J. Yin, Enhancement of prebiotic peptide formation in cyclic environments. *Orig. Life Evol. Biosph.* **53**, 157–173 (2023).

70. R. Osterberg, L. E. Orgel, Polyphosphate and trimetaphosphate formation under potentially prebiotic conditions. *J. Mol. Evol.* **1**, 241–248 (1972).

71. Y. Yamagata, H. Watanabe, M. Saitoh, T. Namba, Volcanic production of polyphosphates and its relevance to prebiotic evolution. *Nature* **352**, 516–519 (1991).

72. A. S. Baidya, M. A. Pasek, E. E. Stüeken, Moderate and high-temperature metamorphic conditions produced diverse phosphorous species for the origin of life. *Commun. Earth Environ.* **5**, 491 (2024).

73. D. Ritson, J. D. Sutherland, Prebiotic synthesis of simple sugars by photoredox systems chemistry. *Nat. Chem.* **4**, 895–899 (2012).

74. O. R. Maguire, I. B. A. Smokers, W. T. S. Huck, A physicochemical orthophosphate cycle via a kinetically stable thermodynamically activated intermediate enables mild prebiotic phosphorylations. *Nat. Commun.* **12**, 5517 (2021).

75. S. J. Zhang, D. Duzdevich, D. Ding, J. W. Szostak, Freeze-thaw cycles enable a prebiotically plausible and continuous pathway from nucleotide activation to nonenzymatic RNA copying. *Proc. Natl. Acad. Sci. U.S.A.* **119**, e2116429119 (2022).

76. J. Oró, B. Basile, S. Cortes, C. Shen, T. Yamrom, The prebiotic synthesis and catalytic role of imidazoles and other condensing agents. *Orig. Life* **14**, 237–242 (1984).

77. C. Gibard, S. Bhowmik, M. Karki, E.-K. Kim, R. Krishnamurthy, Phosphorylation, oligomerization and self-assembly in water under potential prebiotic conditions. *Nat. Chem.* **10**, 212–217 (2018).

78. S. Dixon, M. J. Rutherford, Plagiogranites as late-stage immiscible liquids in ophiolite and mid-ocean ridge suites: An experimental study. *Earth Planet. Sci. Lett.* **45**, 45–60 (1979).

79. J. Ferguson, K. L. Currie, Silicate immiscibility in the ancient "basalts" of the Barberton Mountain Land, Transvaal. *Nat. Phys. Sci.* **235**, 86–89 (1972).

80. M. Coltorti, V. A. V. Girardi, J. H. D. Schorscher, Liquid immiscibility in the Archean Greenstone Belt of Piumhi (Minais Gerais, Brazil). *Lithos* **20**, 77–91 (1987).

81. X. Zeng, K. H. Joy, S. Li, Y. Lin, N. Wang, X. Li, Y. Li, J. Hao, J. Liu, S. Wang, Oldest immiscible Silica-rich Melt on the Moon recorded in a ~4.38 Ga Zircon. *Geophys. Res. Lett.* **47**, e2019GL085997 (2020).

82. F. M. Richter, A major change in the thermal state of the Earth at the Archean-Proterozoic Boundary: Consequences for the nature and preservation of continental lithosphere. *J. Petrol.*, 39–52 (1988).

83. S. O. Agrell, N. R. Charnley, G. A. Chinner, Phosphoran olivine from Pine Canyon, Piute Co., Utah. *Mineral. Mag.* **62**, 265–269 (1998).

84. J. S. Boesenberg, R. H. Hewins, An experimental investigation into the metastable formation of phosphoran olivine and pyroxene. *Geochim. Cosmochim. Acta* **74**, 1923–1941 (2010).

85. Y. Tsukamoto, T. Kakegawa, Phosphate behavior during submarine hydrothermal alteration of ca. 3.455 Ga basaltic seafloor rocks from Pilbara, Western Australia. *Geochim. Cosmochim. Acta* **407**, 224–239 (2025).

86. C. Cheng, C. Fan, R. Wan, C. Tong, Z. Miao, J. Chen, Y. Zhao, Phosphorylation of adenosine with trimetaphosphate under simulated prebiotic conditions. *Orig. Life Evol. Biosph.* **32**, 219–224 (2002).

87. M. Tohidi, L. E. Orgel, Polymerization of the cyclic pyrophosphates of nucleosides and their analogues. *J. Mol. Evol.* **30**, 97–103 (1990).

88. O. R. Maguire, I. B. A. Smokers, B. G. Oosterom, A. Zheliezniak, W. T. S. Huck, A prebiotic precursor to life's phosphate transfer system with an ATP analog and histidyl peptide organocatalysts. *J. Am. Chem. Soc.* **146**, 7839–7849 (2024).

89. Z. Liu, J.-C. Rossi, R. Pascal, How prebiotic chemistry and early life chose phosphate. *Life* **9**, 26 (2019).

90. T. Shea, B. F. Houghton, L. Gurioli, K. V. Cashman, J. E. Hammer, B. J. Hobden, Textural studies of vesicles in volcanic rocks: An integrated methodology. *J. Volcanol. Geotherm. Res.* **190**, 271–289 (2010).

91. D. L. Sahagian, A. A. Proussevitch, 3D particle size distributions from 2D observations: Stereology for natural applications. *J. Volcanol. Geotherm. Res.* **84**, 173–196 (1998).

92. D. Weller, M. Colombier, F. Cáceres, J. Vasseur, D. B. Dingwell, B. Scheu, Confocal scanning laser microscopic (CSLM) characterization of volcanic rocks. *J. Volcanol. Geotherm. Res.* **446**, 107992 (2024).

93. B. Lafuente, R. T. Downs, H. Yang, N. Stone, "1. The power of databases: The RRUFF project," in *Highlights in Mineralogical Crystallography*, T. Armbruster, R. M. Danisi, Eds. (DE GRUYTER, 2015), pp. 1–30.

94. C. R. Lhermitte, N. Plainpan, P. Canjura, F. Boudoire, K. Sivula, Direct photoelectrochemical oxidation of hydroxymethylfurfural on tungsten trioxide photoanodes. *RSC Adv.* **11**, 198–202 (2021).

95. O. Saygin, Non-enzymatic phosphorylation of acetate by carbamyl-phosphate – A model reaction for prebiotic activation of carboxyl groups. *Orig. Life Evol. Biosph.* **13**, 43–48 (1983).

96. J. W. Szostak, The origin of life on Earth and the design of alternative life forms. *Mol. Front. J.* **01**, 121–131 (2017).

97. A. D. Keefe, S. L. Miller, Was ferrocyanide a prebiotic reagent? *Orig. Life Evol. Biosph.* **26**, 111–129 (1996).

98. Z. R. Todd, A. C. Fahrenbach, C. J. Magnani, S. Ranjan, A. Björkbom, J. W. Szostak, D. D. Sasselov, Solvated-electron production using cyanocuprates is compatible with the UV-environment on a Hadean–Archaean Earth. *Chem. Commun.* **54**, 1121–1124 (2018).

99. Y. Yamagata, T. Mohri, Formation of cyanate and carbamyl phosphate by electric discharges of model primitive gas. *Orig. Life* **12**, 41–44 (1982).

100. T. Walton, J. W. Szostak, A highly reactive imidazolium-bridged dinucleotide intermediate in nonenzymatic RNA primer extension. *J. Am. Chem. Soc.* **138**, 11996–12002 (2016).

101. D. K. O'Flaherty, L. Zhou, J. W. Szostak, Nonenzymatic template-directed synthesis of mixed-sequence 3′-NP-DNA up to 25 nucleotides long inside model protocells. *J. Am. Chem. Soc.* **141**, 10481–10488 (2019).

102. J. D. Sutherland, The origin of life—Out of the blue. *Angew. Chem. Int. Ed. Engl.* **55**, 104–121 (2016).

103. M. Halmann, S. Bloch, Glyoxal and malonaldehyde formation by ultraviolet irradiation of aqueous formaldehyde. *Biosystems* **11**, 227–232 (1979).

104. K. Kobayashi, M. Tsuchiya, T. Oshima, H. Yanagawa, Abiotic synthesis of amino acids and imidazole by proton irradiation of simulated primitive earth atmospheres. *Orig. Life Evol. Biosph.* **20**, 99–109 (1990).

105. M. Gull, M. A. Pasek, The role of glycerol and its derivatives in the biochemistry of living organisms, and their prebiotic origin and significance in the evolution of life. *Catalysts* **11**, 86 (2021).

106. B. H. Patel, C. Percivalle, D. J. Ritson, C. D. Duffy, J. D. Sutherland, Common origins of RNA, protein and lipid precursors in a cyanosulfidic protometabolism. *Nat. Chem.* **7**, 301–307 (2015).

107. D. E. Epps, D. W. Nooner, J. Eichberg, E. Sherwood, J. Oró, Cyanamide mediated synthesis under plausible primitive earth conditions: VI. The synthesis of glycerol and glycerophosphates. *J. Mol. Evol.* **14**, 235–241 (1979).

108. Y. Shigemasa, Y. Matsuda, C. Sakazawa, T. Matsuura, Formose reactions. II. The photochemical formose reaction. *Bull. Chem. Soc. Jpn.* **50**, 222–226 (1977).

109. M. Nuevo, J. H. Bredehöft, U. J. Meierhenrich, L. d'Hendecourt, W. H.-P. Thiemann, Urea, glycolic acid, and glycerol in an organic residue produced by ultraviolet irradiation of interstellar/pre-cometary ice analogs. *Astrobiology* **10**, 245–256 (2010).

110. R. I. Kaiser, S. Maity, B. M. Jones, Synthesis of prebiotic glycerol in interstellar ices. *Angew. Chem. Int. Ed. Engl.* **54**, 195–200 (2015).

111. J. R. Cronin, S. Chang, "Organic matter in meteorites: Molecular and isotopic analyses of the Murchison meteorite," in *The Chemistry of Life's Origins*, J. M. Greenberg, C. X. Mendoza-Gómez, V. Pirronello, Eds. (Springer, 1993), pp. 209–258.

112. G. Cooper, N. Kimmich, W. Belisle, J. Sarinana, K. Brabham, L. Garrel, Carbonaceous meteorites as a source of sugar-related organic compounds for the early Earth. *Nature* **414**, 879–883 (2001).

113. F. Wöhler, Ueber künstliche Bildung des Harnstoffs. *Ann. Phys.* **88**, 253–256 (1828).

114. C. U. Lowe, M. W. Rees, R. Markham, Synthesis of complex organic compounds from simple precursors: Formation of amino-acids, amino-acid polymers, fatty acids and purines from ammonium cyanide. *Nature* **199**, 219–222 (1963).

115. K. G. Clark, V. L. Gaddy, C. E. Rist, Equilibria in the ammonium carbamate-urea-water system. *Ind. Eng. Chem.* **25**, 1092–1096 (1933).

116. S. L. Miller, The mechanism of synthesis of amino acids by electric discharges. *Biochim. Biophys. Acta* **23**, 480–489 (1957).

117. M. J. Bishop, R. Lohrmann, L. E. Orgel, Prebiotic phosphorylation of thymidine at 65°C in simulated desert conditions. *Nature* **237**, 162–164 (1972).

118. B. Burcar, M. Pasek, M. Gull, B. J. Cafferty, F. Velasco, N. V. Hud, C. Menor-Salván, Darwin's warm little pond: A one-pot reaction for prebiotic phosphorylation and the mobilization of phosphate from minerals in a urea-based solvent. *Angew. Chem. Int. Ed. Engl.* **55**, 13249–13253 (2016).

119. M. Gull, M. Zhou, F. M. Fernández, M. A. Pasek, Prebiotic phosphate ester syntheses in a deep eutectic solvent. *J. Mol. Evol.* **78**, 109–117 (2014).

120. G. Cooper, C. Reed, D. Nguyen, M. Carter, Y. Wang, Detection and formation scenario of citric acid, pyruvic acid, and other possible metabolism precursors in carbonaceous meteorites. *Proc. Natl. Acad. Sci. U.S.A.* **108**, 14015–14020 (2011).

121. R. T. Stubbs, M. Yadav, R. Krishnamurthy, G. Springsteen, A plausible metal-free ancestral analogue of the Krebs cycle composed entirely of α-ketoacids. *Nat. Chem.* **12**, 1016–1022 (2020).

122. G. Springsteen, J. R. Yerabolu, J. Nelson, C. J. Rhea, R. Krishnamurthy, Linked cycles of oxidative decarboxylation of glyoxylate as protometabolic analogs of the citric acid cycle. *Nat. Commun.* **9**, 91 (2018).

123. I. B. A. Smokers, M. H. I. Van Haren, T. Lu, E. Spruijt, Complex coacervation and compartmentalized conversion of prebiotically relevant metabolites. *ChemSystemsChem* **4**, e202200004 (2022).

124. M. Gull, M. A. Mojica, F. M. Fernández, D. A. Gaul, T. M. Orlando, C. L. Liotta, M. A. Pasek, Nucleoside phosphorylation by the mineral schreibersite. *Sci. Rep.* **5**, 17198 (2015).
